# An integrated chemokine-based machine learning model predicts prognosis and guides immunotherapy in hepatocellular carcinoma

**DOI:** 10.1097/MD.0000000000046586

**Published:** 2025-12-19

**Authors:** Jinlong Zhou, Junjie Liu, Xinrong Wei, Cheng Liu, Hao Gu

**Affiliations:** a Department of Liver Transplantation and Laparoscopic Surgery, The First Affiliated Hospital of Xinjiang Medical University, Urumqi, China; b Department of Hepatobiliary Surgery, Eastern Hepatobiliary Surgery Hospital, Navy Medical University, ShanghaiChina; c Department of Liver Transplantation and Laparoscopic Surgery, The First Affiliated Hospital of Xinjiang Medical University, Urumqi, China.

**Keywords:** chemokines, hepatocellular carcinoma, immunotherapy response, machine learning, tumor microenvironment

## Abstract

Hepatocellular carcinoma (HCC), the second leading cause of cancer-related death globally, has limited clinical benefits from immune checkpoint inhibitors. This study aimed to address the critical challenges of prognostic heterogeneity and low immunotherapy response rates in patients with HCC by integrating chemokine-related gene expression profiles using Machine learning (ML) algorithms. Using the cancer genome atlas hepatocellular carcinoma and GSE14520 datasets, we performed unsupervised consensus clustering of 227 chemokine-related genes to define HCC subtypes. Prognostic genes were screened using univariate Cox regression, and 10 ML algorithms were integrated under 10-fold cross-validation to construct a predictive model. Immune infiltration, pathway enrichment, and immunotherapy sensitivity were analyzed using ESTIMATE, single-sample gene set enrichment analysis, and immunophenoscore. Two chemokine-related subtypes (A/B) were identified, and subtype B demonstrated a significantly prolonged overall survival (*P* < .05). The StepCox[both] + SuperPC model, constructed from 92 prognostic genes, exhibited robust performance in both training and validation sets (*C*-index: 0.719/0.653). High-risk patients were characterized by metabolic reprogramming and an immunosuppressive tumor microenvironment, whereas the low-risk group displayed immune-stromal synergy. Among the 11 core genes screened, high expression of SPP1 and SLC1A2 was significantly associated with poor prognosis (*P* < .05), whereas ITGAM/HILPDA may serve as a predictor of programmed cell death protein 1/cytotoxic T-lymphocyte-associated protein 4 inhibitor sensitivity. In this study, we developed a chemokine-based ML model that classifies HCC patients into 2 subtypes with distinct survival outcomes and immune-metabolic features. We identified an 11-gene prognostic signature, including SPP1 and SLC2A1 associated with poor prognosis, and genes such as ITGAM and P2RY6 predictive of immunotherapy response. These findings provide insights into the chemokine-immune-metabolic network and support further validation toward personalized HCC treatment.

## 1. Introduction

Hepatocellular carcinoma (HCC), the seventh most common malignant tumor globally and the second leading cause of cancer-related deaths, has increased the disease burden. Epidemiological data predict that HCC-related mortality will exceed 1 million by 2030^[1,2]^ Although systemic therapies involving immune checkpoint inhibitors (ICIs) have revolutionized treatment paradigms for advanced HCC, significant challenges persist in achieving durable survival benefits. Compared with sorafenib, the 2020 IMbrave150 phase III clinical trial demonstrated that the combination of atezolizumab (an anti-PD-L1 monoclonal antibody) and bevacizumab (an antiVEGF monoclonal antibody) significantly improved the median overall survival (OS), progression-free survival, and objective response rate.^[3,4]^ Subsequent phase III trials (the STRIDE regimen and camrelizumab combined with rivoceranib) further validated the therapeutic superiority of ICI-based combination therapies, establishing them as new first-line standards for unresectable HCC.^[5–9]^ However, in clinical practice, only 15% to 30% of patients achieve sustained therapeutic responses,^[7]^ underscoring the urgent need for precise identification of immunotherapy-benefiting populations.

As pivotal molecular regulators of the tumor microenvironment (TME), chemokines warrant in-depth exploration of their potential value as biomarkers. This protein family not only mediates the directional migration of immune cells such as monocytes and dendritic cells through ligand–receptor-specific binding^[10]^ but also directly modulates tumor proliferation, angiogenesis, and metastatic processes by acting on tumor cells and vascular endothelial cells.^[11]^ Notably, specific chemokines dynamically reshape the immunosuppressive state of the TME through differential recruitment of immune cell subsets, thereby influencing the intensity of antitumor immune responses.^[4,12]^ For instance, upregulation of the C-X-C motif chemokine ligand (CXCL) 1 axis promotes anti- programmed cell death protein 1 (PD-1) resistance by facilitating tumor-associated neutrophil infiltration in HCC.^[13]^ CXCL12^+^ tumor-associated endothelial cells have been shown to inhibit the differentiation of CD8^+^ native T cells into cytotoxic T cells and recruit myeloid-derived suppressor cells, thereby suppressing the efficacy of immunotherapy in HCC.^[14]^ Additionally, disruption of C-C motif chemokine ligand (CCL)2 signaling and macrophage M2 polarization following SLFN11 deficiency enhances the efficacy of anti-PD-1 therapy.^[15]^ Protease PRSS35 can suppress HCC progression by cleaving CXCL2 and inhibiting neutrophil extracellular traps.^[16]^ Furthermore, CCL5 facilitates immune evasion of circulating tumor cells via the p38–MAX signaling pathway, recruiting regulatory T cells to promote immune escape and metastatic seeding.^[17]^ Alternatively, by binding specific receptors, CCL5 inhibits the ubiquitination and degradation of hypoxia-inducible factor 1 alpha, maintaining its expression under normoxic conditions and upregulating zinc finger E-box binding homeobox 1 to induce epithelial–mesenchymal transition (EMT), thereby promoting pulmonary metastasis of HCC.^[18]^ This multidimensional regulatory mechanism suggests that the chemokine network may serve as a potential molecular target for predicting ICI efficacy by integrating immune cell infiltration characteristics with tumor biological behavior.

Rapid advancements in artificial intelligence technology have led to novel paradigms for deciphering complex biomarker networks. Machine learning (ML), which employs algorithms such as supervised learning, unsupervised learning, and reinforcement learning, has unique advantages in tumor prognosis prediction and therapeutic response stratification through autonomous identification of latent patterns in high-dimensional data.^[19,20]^ Ensemble learning strategies based on ML enhance the robustness of heterogeneous HCC biomarker screening by integrating multi-model predictions, effectively mitigating single-algorithm bias, and providing methodological support for establishing chemokine-related predictive models.

This study aimed to systematically screen chemokine biomarkers closely associated with HCC immunotherapy response through the development of a robust ensemble ML-based framework while constructing interpretable predictive models. The research outcomes are expected to provide theoretical foundations for personalized treatment strategies for patients with HCC and offer methodological references for multidimensional integrative analysis of biomarkers in cancer immunotherapy.

## 2. Materials and methods

### 2.1. Data acquisition

This study retrieved the mRNA expression profiles and clinical data of patients with HCC from the cancer genome atlas hepatocellular carcinoma (TCGA-LIHC, https://portal.gdc.cancer.gov/) and the Gene Expression Omnibus (GSE14520, https://www.ncbi.nlm.nih.gov/geo/). The inclusion criteria were available mRNA expression data and complete clinical information. Batch effects between the TCGA-LIHC and GSE14520 datasets were mitigated using the ComBat algorithm (R package sva). Chemokine-related genes were retrieved from the Molecular Signatures Database and GeneCards, and duplicate genes were removed for downstream analyses.

### 2.2. Construction of chemokine-based HCC subtypes

To identify chemokine-related subtypes in HCC, unsupervised consensus clustering was performed on chemokine-related genes using R package ConsensusClusterPlus. Prior to clustering, differentially expressed genes (DEGs) between tumor tissues and adjacent non-tumor tissues were identified using the R package limma, and chemokine-related DEGs were selected for clustering. Clustering stability was evaluated using cumulative distribution function plots and consensus matrix heatmaps.

### 2.3. Characterization of subtypes and immune landscapes

To explore the biological heterogeneity among subtypes, DEGs between subtypes were screened (|log_2_ FC| > 1, false discovery rate < 0.05). Gene ontology (GO) and kyoto encyclopedia of genes and genomes (KEGG) pathway enrichment analyses were conducted via the DAVID database to elucidate potential biological functions and pathways.

### 2.4. Integrated machine learning for core gene screening

Prognostic gene identification Univariate Cox regression analysis was applied to subtype-specific DEGs in the TCGA-LIHC cohort to identify prognosis-associated genes. Model development and validation: The TCGA-LIHC dataset served as the training set and GSE14520 served as the validation set. we utilized 10 algorithms – Lasso, Ridge, Stepwise Cox, CoxBoost, RSF, Enet, plsRcox, SuperPC, GBM, and Survival-SVM – under 10-fold cross-validation to develop optimal models for predicting patient prognosis based on the input variables and provided cohorts.^[21]^ The models were ranked using the mean Harrell’s concordance index (*C*-index) across the training and validation sets, with the optimal algorithms selected. Risk stratification and survival analysis: Patients were dichotomized into high- and low-risk groups based on median risk scores. Kaplan–Meier curves with log-rank tests were used to compare OS, while receiver operating characteristic (ROC) curves were used to assess the accuracy of 1-, 3-, and 5-year survival prediction.

### 2.5. Tumor microenvironment and immunological profiling

Core gene validation: The prognostic performance of the core genes was validated using survival analysis and ROC curves. Immune infiltration analysis: The ESTIMATE algorithm was used to quantify immune scores, and single-sample gene set enrichment analysis was used to evaluate immune cell infiltration differences between the risk groups. Immunotherapy response prediction: Immunophenoscore (IPS) was used to assess sensitivity to immunotherapy (PD-1 inhibitors, cytotoxic T-lymphocyte-associated protein 4 (CTLA-4) inhibitors, and combination therapy). ICI responses were categorized into 4 groups: CTLA4^+^/PD1^+^, CTLA4^+^/PD1^−^, CTLA4^−^/PD1^+^, and CTLA4^−^/PD1^−^.

### 2.6. Statistical analysis

Data management and statistical analyses were performed using R (v4.2.1) and GraphPad Prism (v8.3.0), respectively. A 2-tailed *P* value < .05 was considered statistically significant. Chi-square and Wilcoxon tests were used to compare clinical characteristics between the training and validation sets. False discovery rate-adjusted *P*-values were used for DEG analysis. K–M survival analysis (R package survival), log-rank tests, and Cox regression were used to identify independent prognostic factors. Time-dependent ROC analysis (R package timeROC) was used to calculate the area under the curve (AUC) values to evaluate the model performance.

## 3. Results

### 3.1. Identification of chemokine-related subtypes in HCC

**Figure 1.**
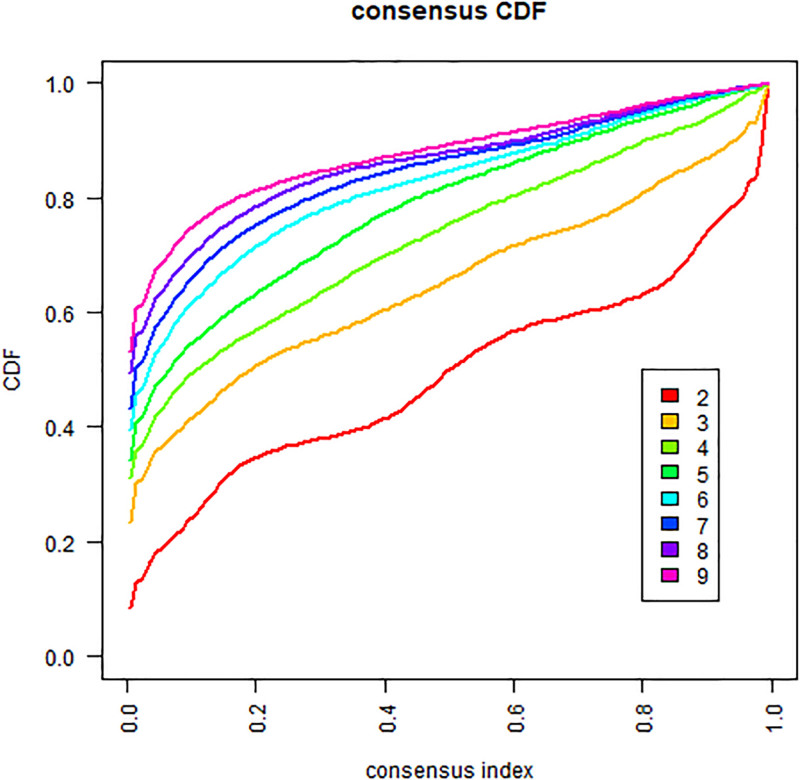
Cumulative distribution function (CDF) plot from consensus clustering analysis of chemokine-related genes in HCC. The curve demonstrates the stability of cluster assignments across multiple iterations, supporting the selection of *k* = 2 as the optimal number of subtypes. CDF = cumulative distribution function, HCC = hepatocellular carcinoma.

**Figure 2.**
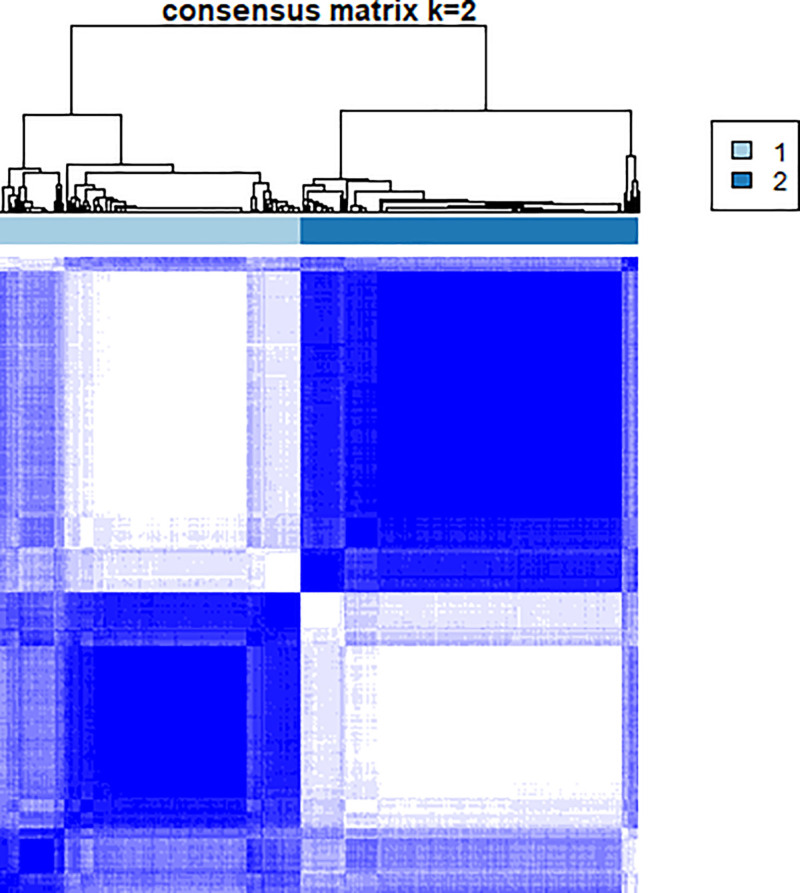
Consensus matrix heatmap for *k* = 2, illustrating the sample-wise clustering stability. Each cell represents the proportion of iterations in which 2 samples were grouped together, with darker blue indicating higher consensus. The clear block structure confirms robust separation between the 2 subtypes.

**Figure 3.**
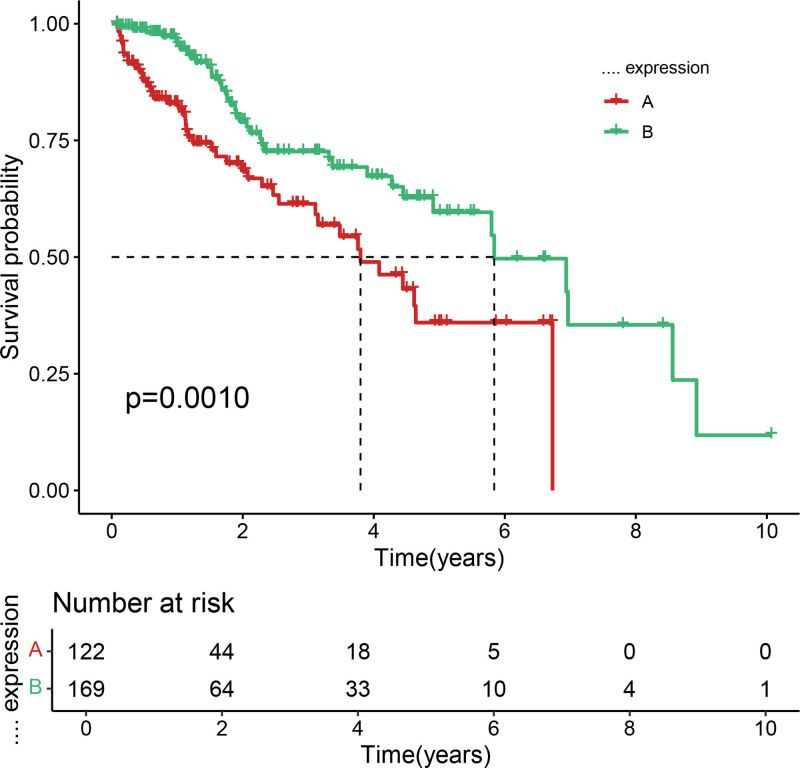
Kaplan–Meier survival curves comparing OS between chemokine-based HCC subtypes A and B in the TCGA cohort. Subtype B shows significantly prolonged survival compared to subtype A (*P* < .05). HCC = hepatocellular carcinoma, OS = overall survival, TCGA = the cancer genome atlas.

**Figure 4.**
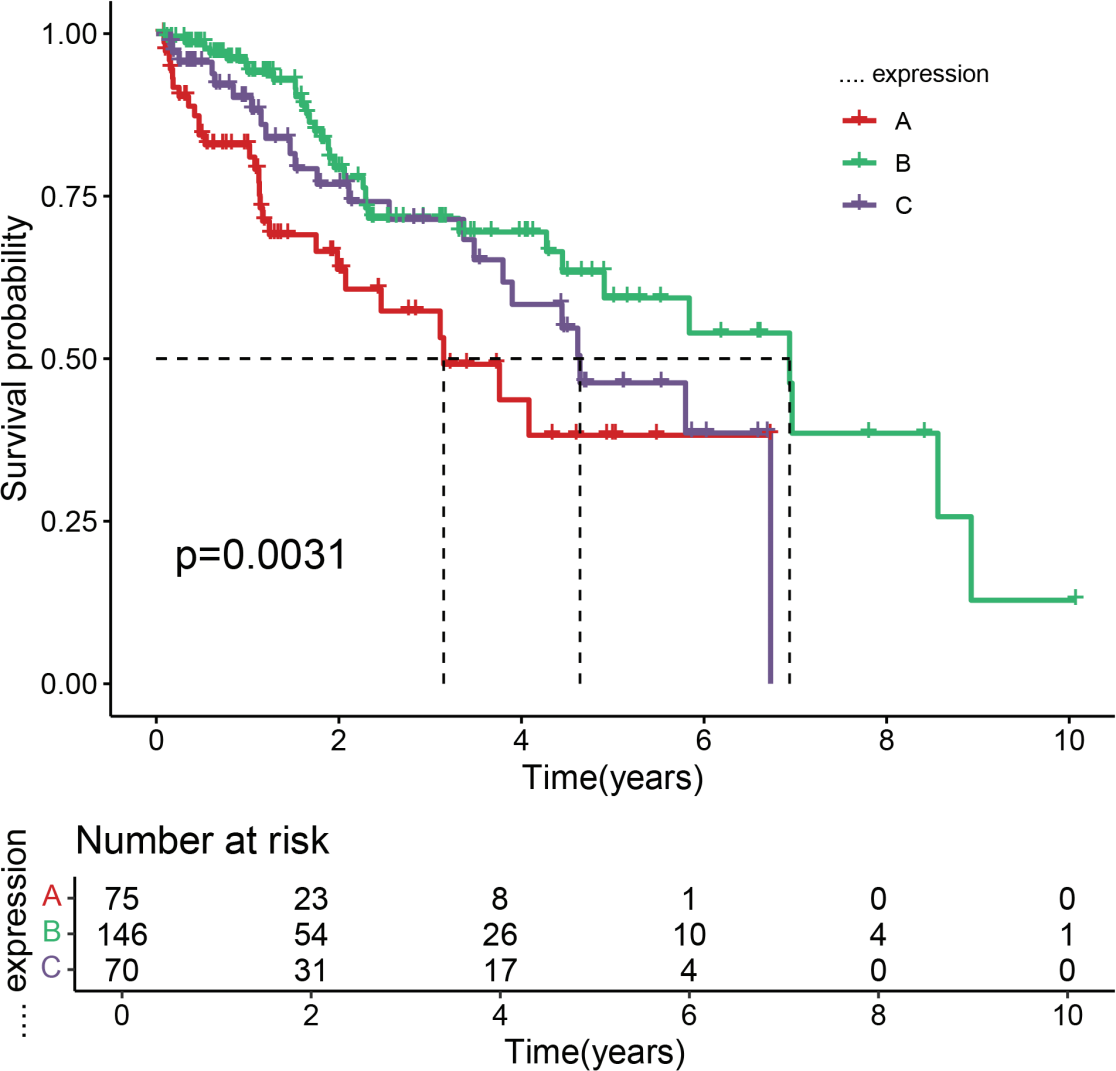
survival analysis of HCC samples based on chemokine-related genes for *k* = 3. Kaplan–Meier survival curves demonstrating significant differences in overall survival among the 3 clusters (*P* < .05), though with less distinct separation compared to *k* = 2. HCC = hepatocellular carcinoma.

**Figure 5.**
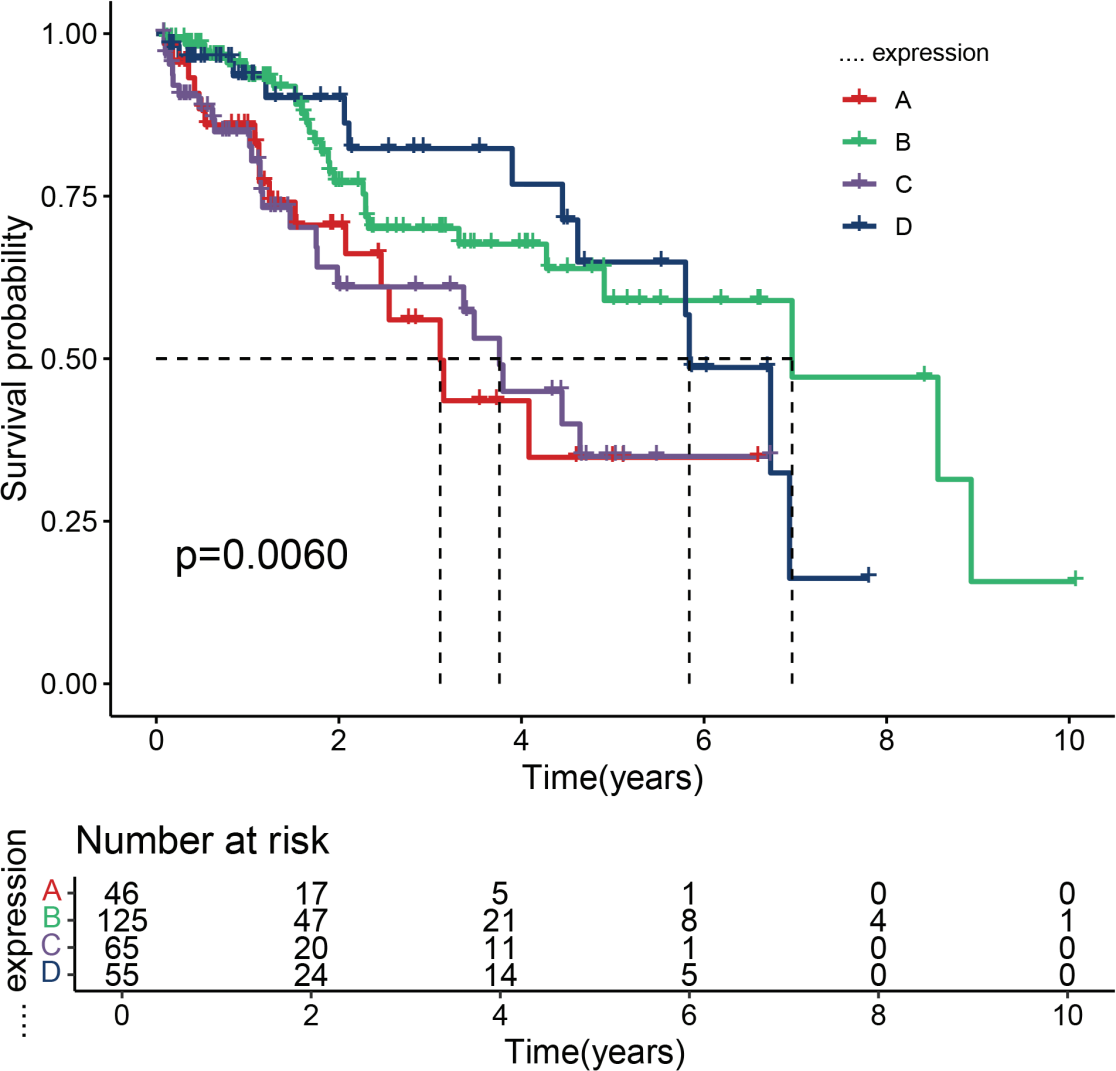
Survival analysis of HCC samples based on chemokine-related genes for *k* = 4. Kaplan–Meier survival analysis revealing statistically significant survival differences among the 4 clusters (*P* < .05), albeit with overlapping survival trends and less clinical interpretability than *k* = 2. HCC = hepatocellular carcinoma.

After filtering, 227 chemokine-related genes were selected for unsupervised consensus clustering analysis. Multiple cluster numbers (*k* = 2–10) were evaluated. The optimal clustering stability and most pronounced prognostic separation were achieved at *k* = 2, as evidenced by the cumulative distribution function plots (Fig. 1) and consensus matrix heatmaps (Fig. 2). All samples were robustly classified into 2 clusters with low intergroup correlations. Survival analysis revealed a significantly shorter OS in cluster A than in cluster B (*P* < .05; Fig. 3). Although *k* = 3 (Fig. 4) and *k* = 4 (Fig. 5) also yielded statistically significant survival differences between subgroups, the separation was less distinct compared to *k* = 2.

### 3.2. Biological functions and immune landscapes of HCC subtypes

**Figure 6.**
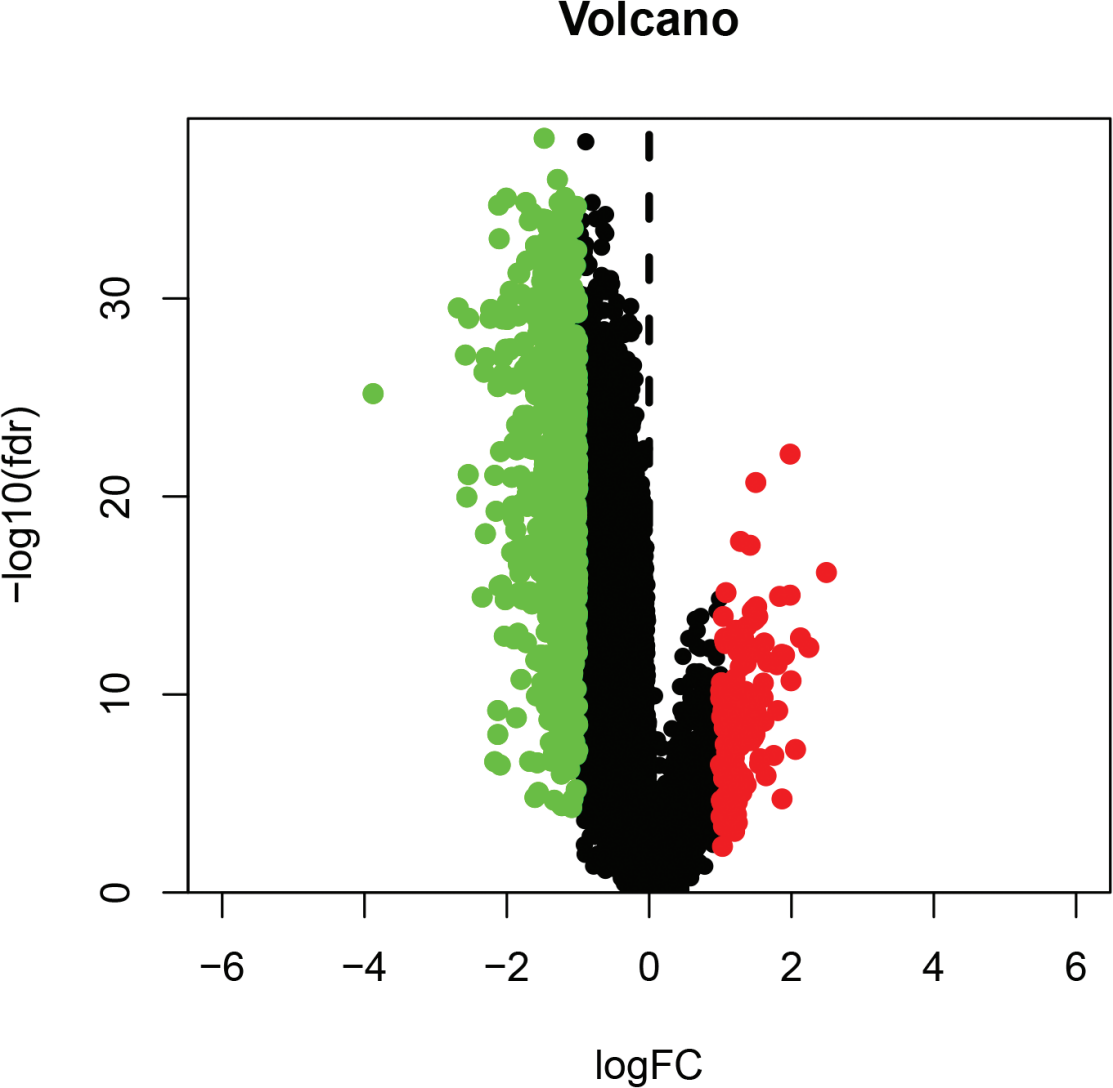
Volcano plot of differentially expressed genes (DEGs) between subtypes A and B. Points represent genes, with red indicating significantly up-regulated genes and blue indicating down-regulated genes in subtype B relative to subtype A. DEG = differentially expressed gene.

**Figure 7.**
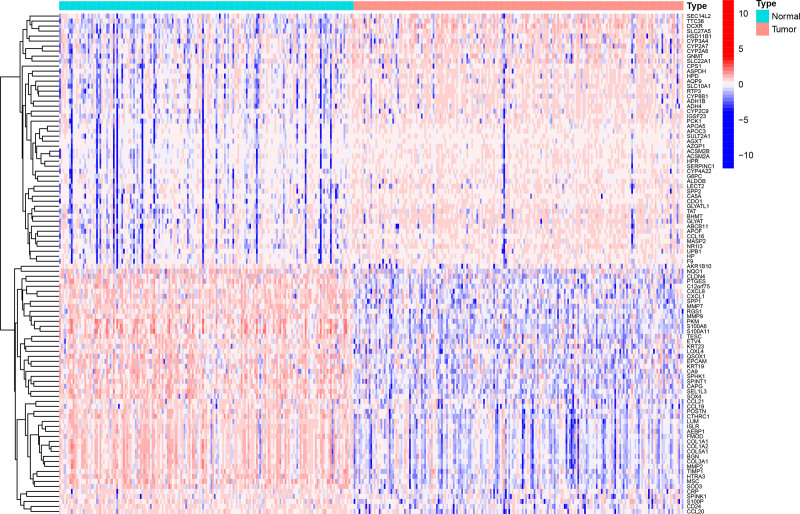
Heatmap of differentially expressed genes between subtypes A and B.

**Figure 8.**
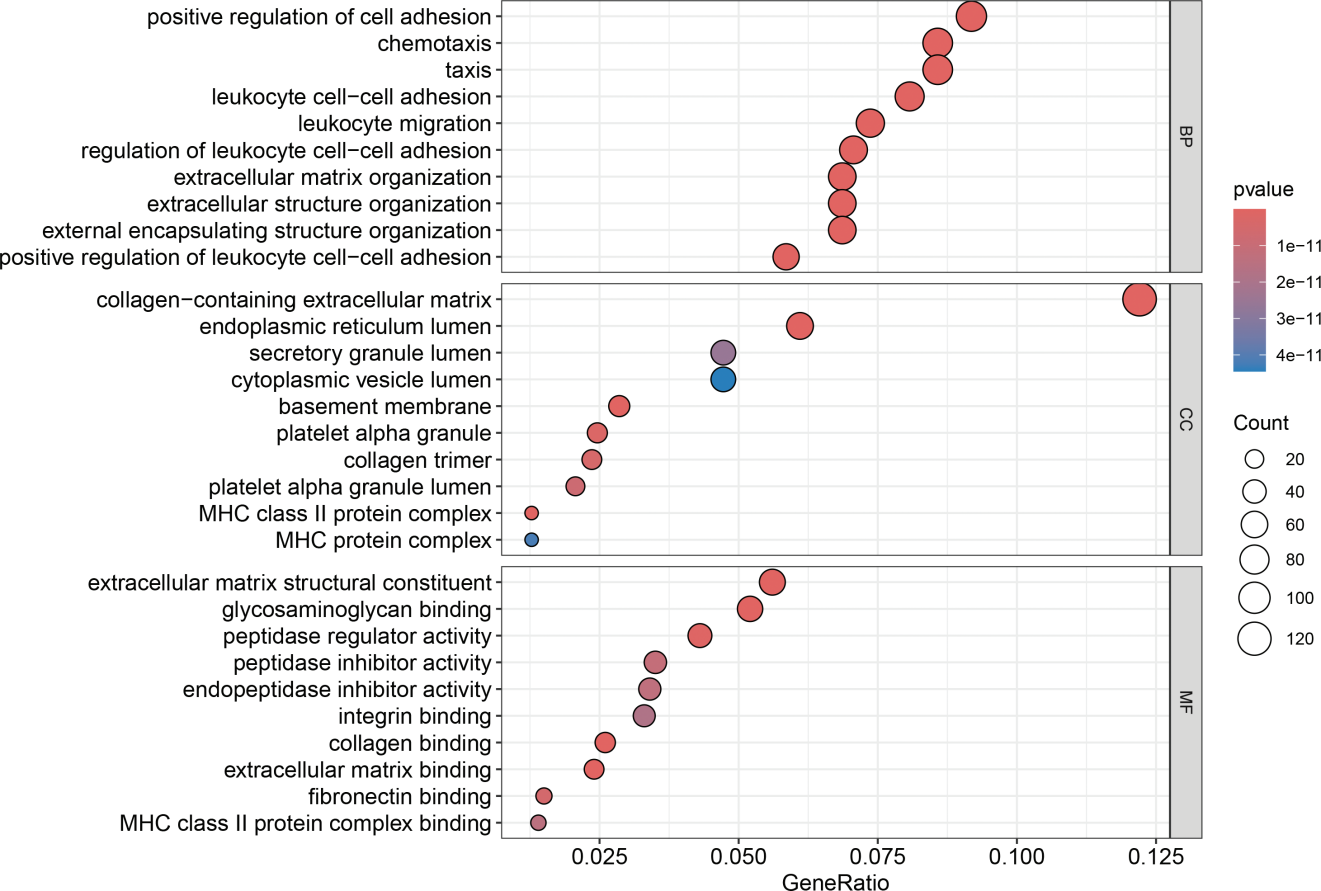
Bubble plot of GO enrichment analysis for DEGs between subtypes A and B. Bubble size represents the number of genes, and color indicates the adjusted *P*-value. DEG = differentially expressed gene, GO = gene ontology.

**Figure 9.**
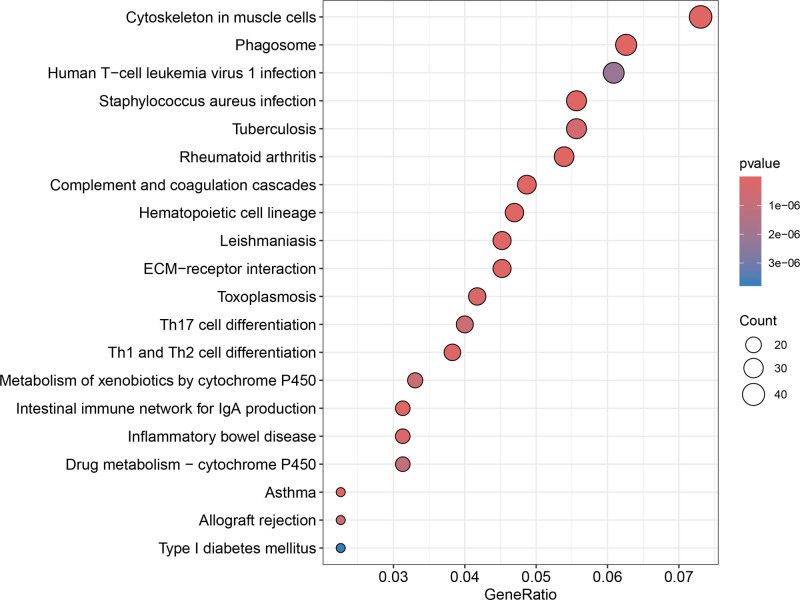
Bubble plot of KEGG pathway enrichment analysis for DEGs between subtypes A and B. Significantly enriched pathways include Th cell differentiation, ECM-receptor interaction, and xenobiotic metabolism. DEG = differentially expressed gene, ECM = extracellular matrix, KEGG = kyoto encyclopedia of genes and genomes.

Differential expression analysis revealed 1032 significant DEGs between the clusters (Figs. 6 and 7). GO enrichment highlighted processes including leukocyte migration, chemotaxis, extracellular matrix (ECM) organization, ECM structural constituents, and cytochrome P450-mediated drug metabolism (Fig. 8). KEGG pathway analysis revealed enrichment in Th17/Th1/Th2 cell differentiation, ECM-receptor interaction, phagosomes, tuberculosis, toxoplasmosis, inflammatory bowel disease, rheumatoid arthritis, and xenobiotic metabolism (Fig. 9). These findings suggest that chemokine-related genes modulate immune cell trafficking, TME remodeling, infection/inflammatory responses, and drug metabolism.

### 3.3. Prognostic signature construction via ensemble machine learning

**Figure 10.**
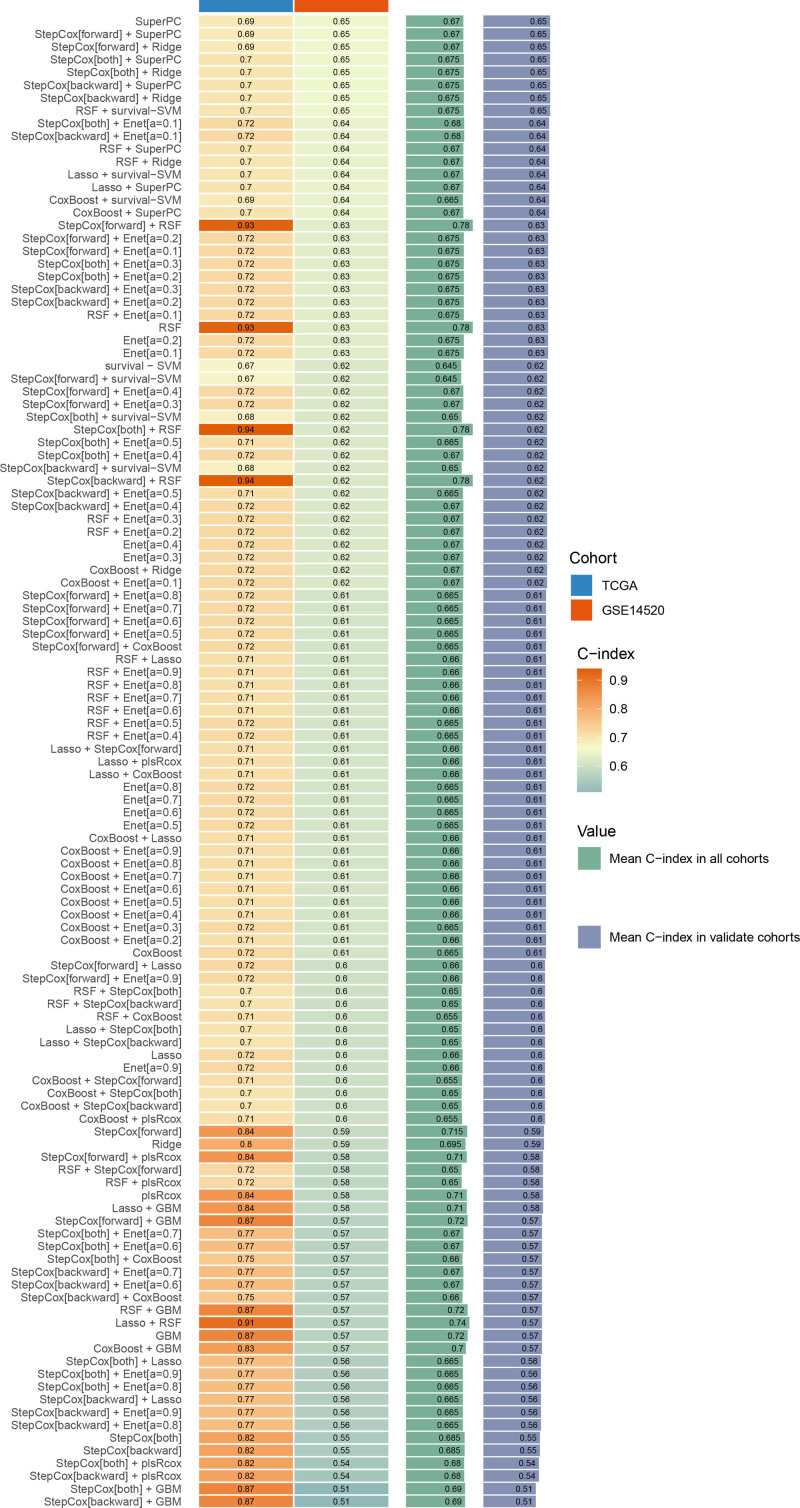
Performance evaluation of 101 prediction models built using 10 machine learning algorithms under 10-fold cross-validation. Models are ranked by mean C-index across validation sets.

**Figure 11.**
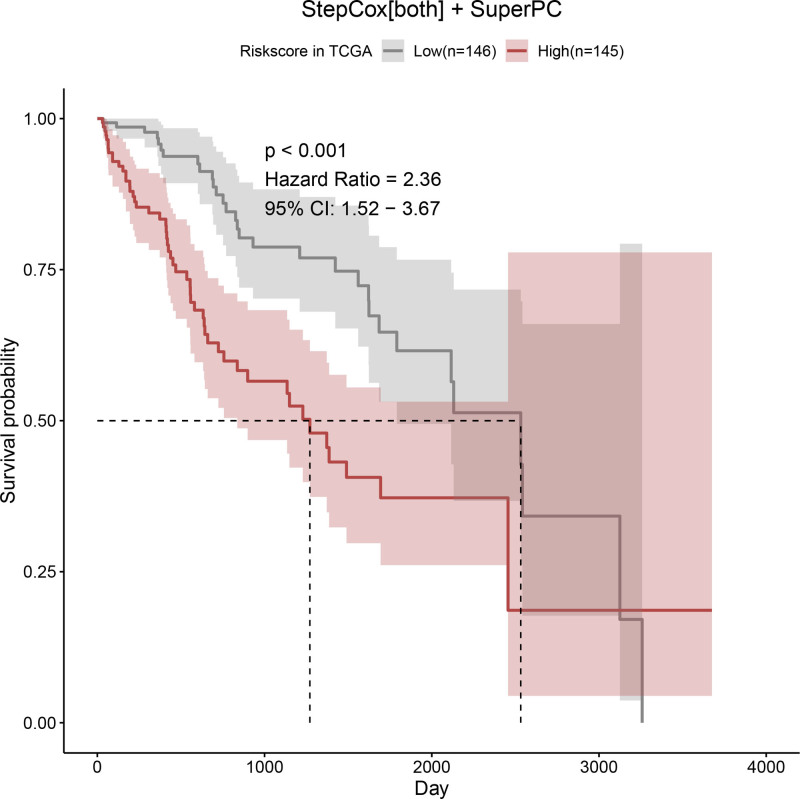
Kaplan–Meier survival curves for OS between high- and low-risk groups in the TCGA training cohort, stratified by median risk score. The high-risk group shows significantly worse survival (*P* < .001). OS = overall survival, TCGA = the cancer genome atlas.

**Figure 12.**
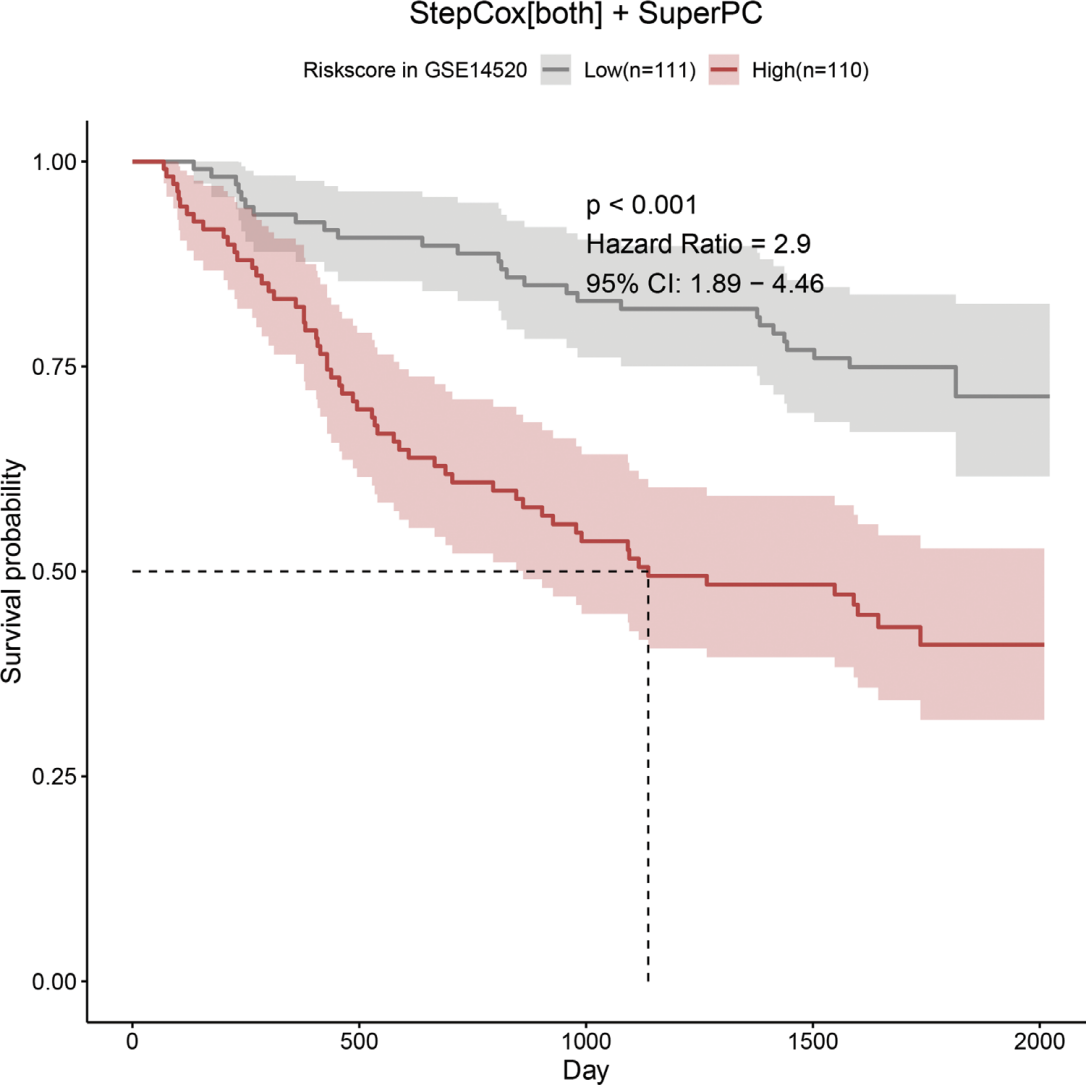
K–M survival curves for OS between high- and low-risk groups in the GSE14520 validation cohort. Consistent with the training set, the high-risk group exhibits poorer survival (*P* < .001). OS = overall survival, K–M = Kaplan–Meier.

**Figure 13.**
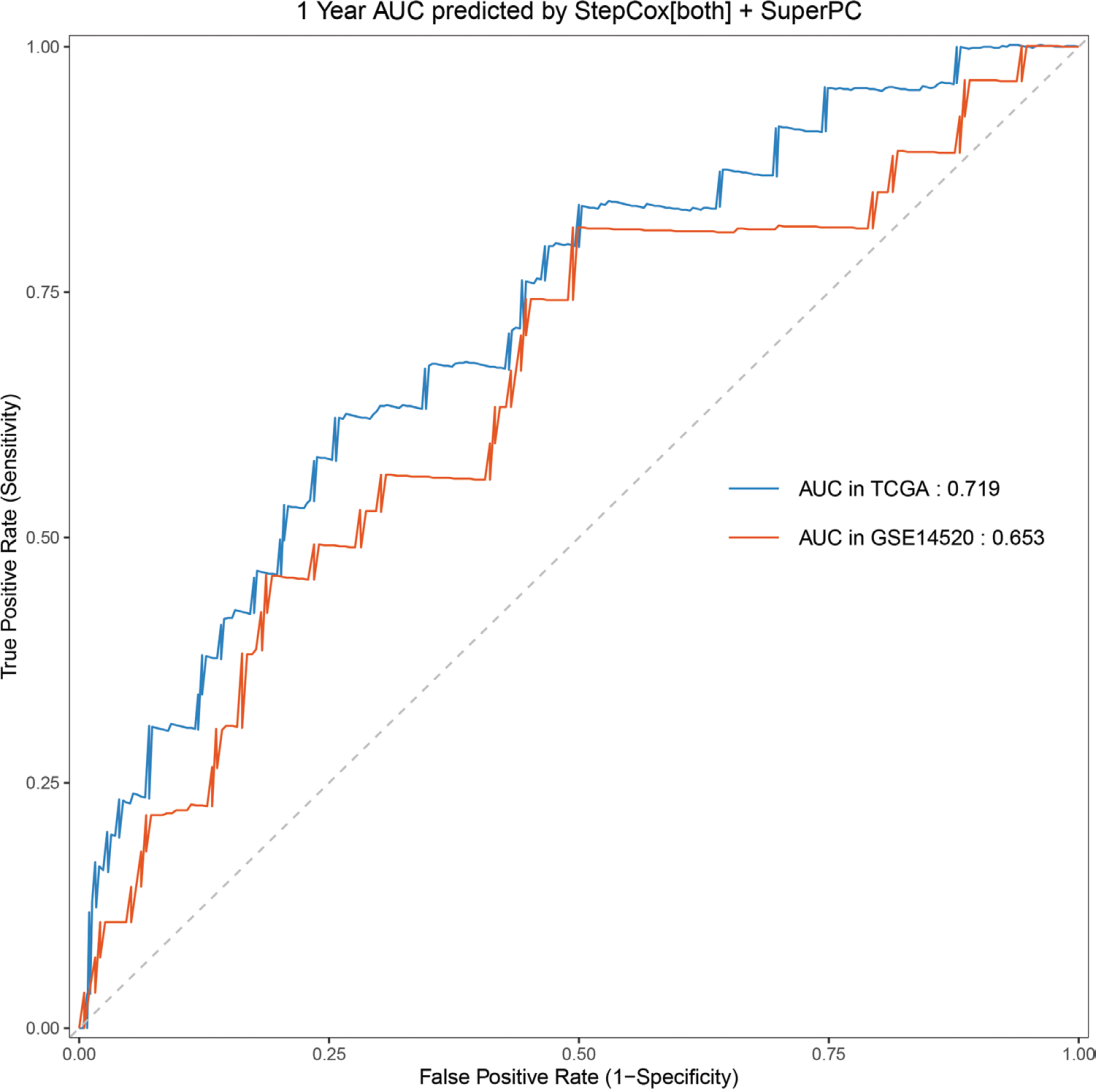
Time-dependent ROC curves for 1-yr OS prediction in the high- and low-risk groups from both TCGA and GSE14520 cohorts. In the training set (TCGA), sensitivity and specificity were 62.2% and 74.4%, respectively; in the validation set (GSE14520), sensitivity and specificity were 81.6% and 50.6%. OS = overall survival, ROC = receiver operating characteristic, TCGA = the cancer genome atlas.

**Figure 14.**
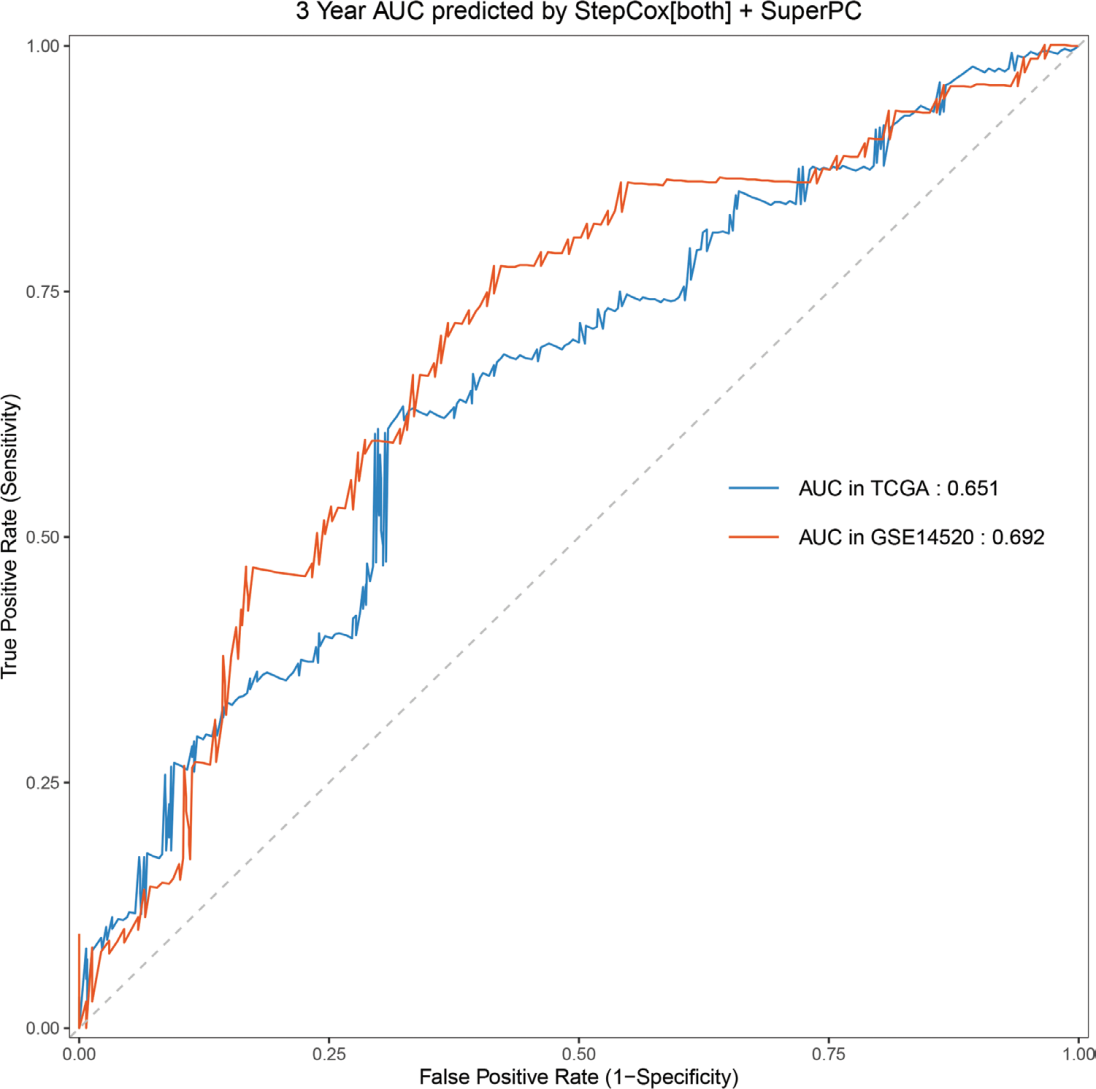
Time-dependent ROC curves for 3-yr OS prediction in the high- and low-risk groups. Sensitivity and specificity were 61.0% and 70.1% in the training set, and 77.6% and 58.5% in the validation set. OS = overall survival, ROC = receiver operating characteristic.

**Figure 15.**
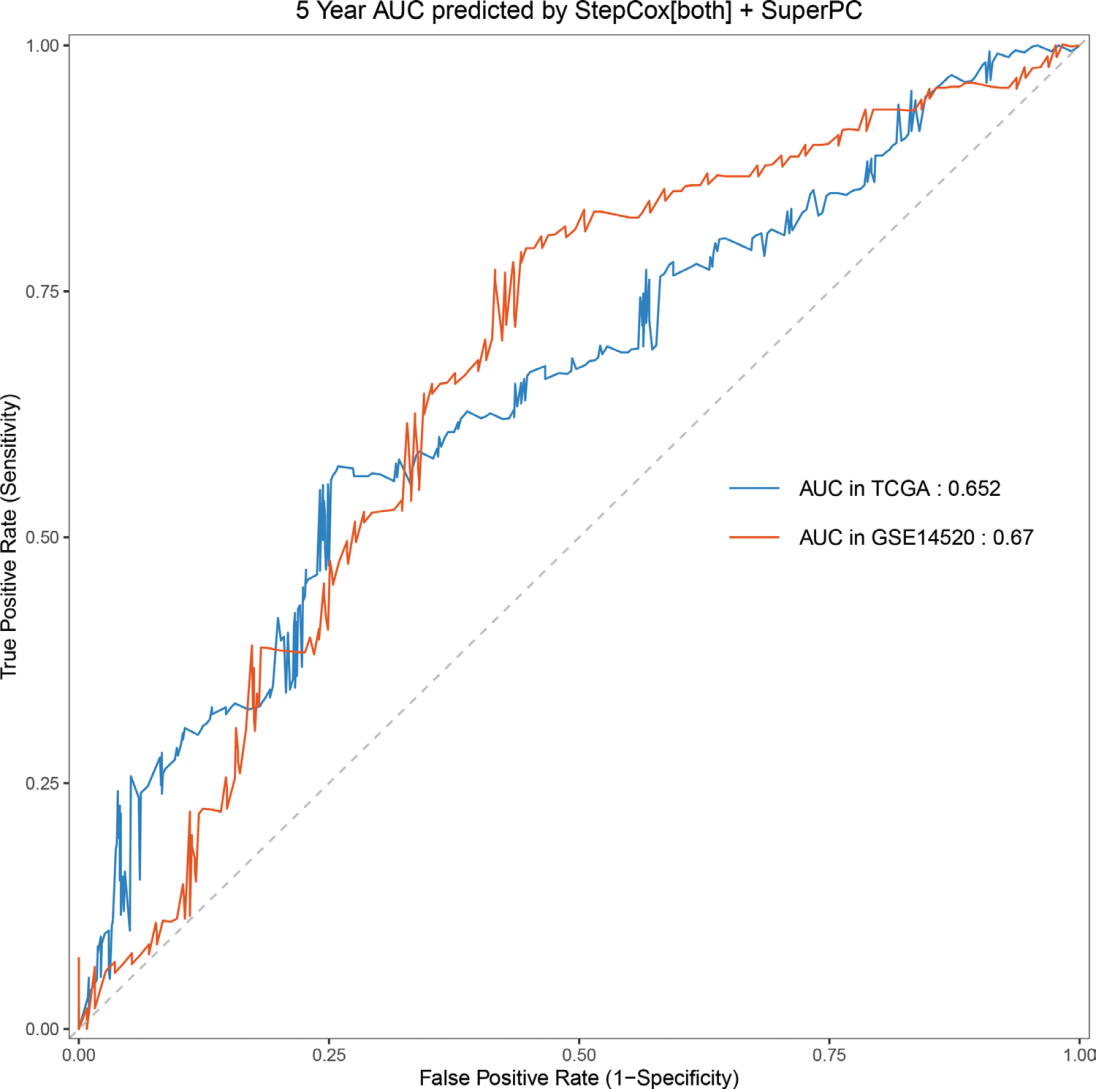
Time-dependent ROC curves for 5-yr OS prediction in the high- and low-risk groups. Sensitivity and specificity were 56.7% and 74.3% in the training set, and 77.2% and 58.4% in the validation set. OS = overall survival, ROC = receiver operating characteristic.

**Figure 16.**
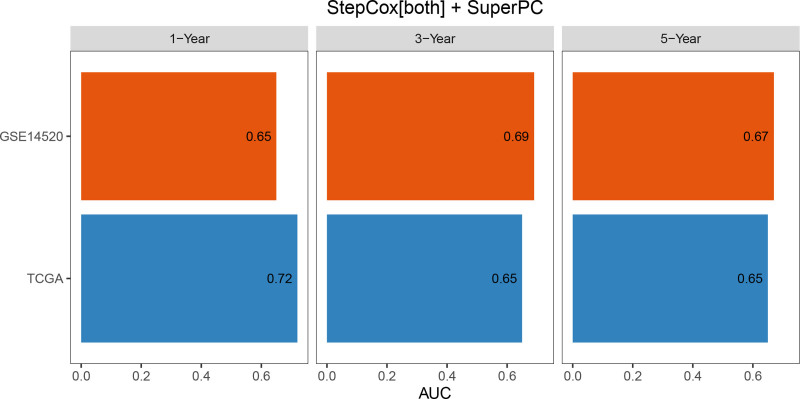
Comparative performance of the prognostic model over time in the training and validation cohorts. This summary bar plot compares the AUC values for predicting 1-, 3-, and 5-yr OS. The model demonstrates consistent and robust predictive performance across both the TCGA (training) and GSE14520 (validation) datasets over all 3 time points. AUC = area under the curve, OS = overall survival, TCGA = the cancer genome atlas.

**Figure 17.**
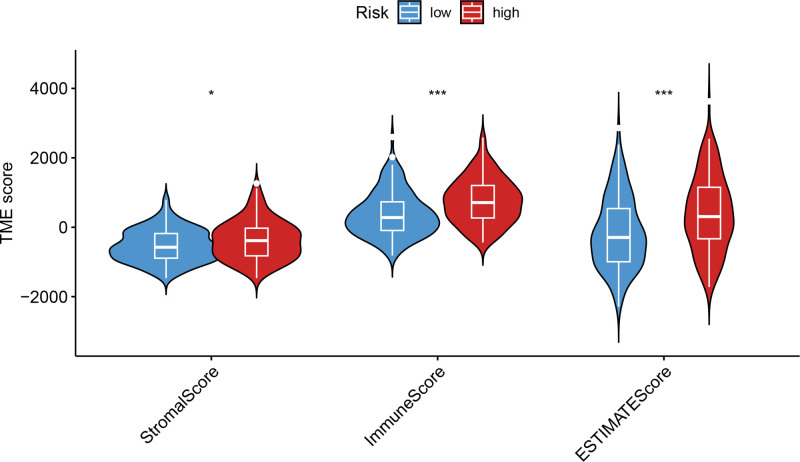
Violin plots comparing ESTIMATE immune and stromal scores between high- and low-risk groups. High-risk patients show significantly higher immune scores but similar stromal scores.

**Figure 18.**
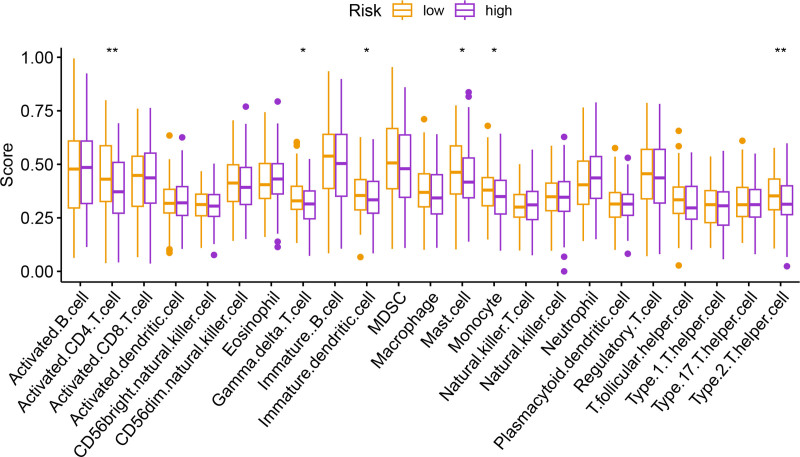
ssGSEA-derived infiltration scores of 23 immune cell types in high- and low-risk groups. Notable differences are observed in activated CD4+ T cells and Th2 cells. ssGSEA = single-sample gene set enrichment analysis.

**Figure 19.**
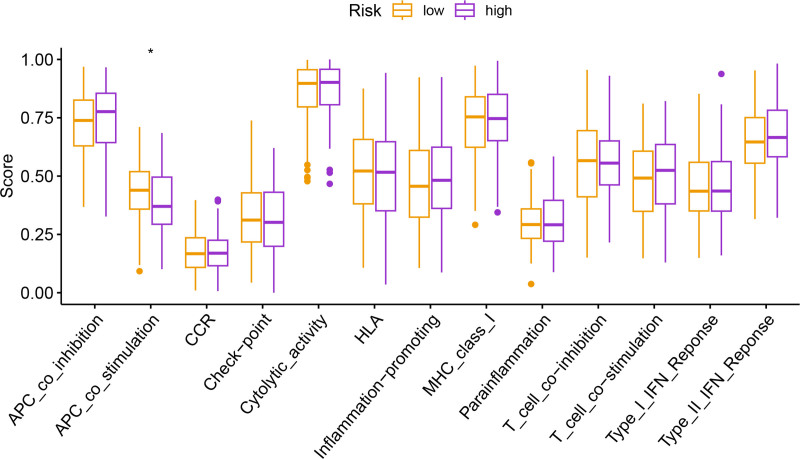
Box plots showing activity levels of 13 immune-related pathways between risk groups. Low-risk patients exhibit enhanced antigen-presenting cell co-stimulation.

Univariate Cox regression analysis revealed 92 prognosis-associated chemokine-related genes. Using TCGA-LIHC data as the training set and GSE14520 data as the validation set, 101 prediction models were evaluated via 10-fold cross validation. The StepCox[both] + SuperPC algorithm demonstrated the highest concordance index and was selected as the final model (Fig. 10). Patients stratified into high- and low-risk groups by median risk scores exhibited significantly shorter OS in the high-risk group across both datasets (*P* < .001; Figs. 11 and 12). Time-dependent ROC analysis revealed AUCs of 0.719, 0.651, and 0.652 (training set), and 0.653, 0.692, and 0.670 (validation set) for 1-, 3-, and 5-year survival, respectively (Figs. 13–16). To further evaluate the clinical utility of the risk model, the optimal cutoff value was determined using the Youden Index. In the training set, the sensitivity and specificity were 62.2% and 74.4% for 1-year survival, 61.0% and 70.1% for 3-year survival, and 56.7% and 74.3% for 5-year survival, respectively. In the validation set, the sensitivity and specificity were 81.6% and 50.6% for 1-year survival, 77.6% and 58.5% for 3-year survival, and 77.2% and 58.4% for 5-year survival, respectively. These results indicate that the model exhibits higher sensitivity in the external validation set, effectively identifying true high-risk patients, while maintaining moderate specificity. Immune infiltration analysis via ESTIMATE revealed higher immune scores in high-risk patients (Fig. 17), whereas low-risk patients presented increased infiltration of activated CD4^+^ T cells and Th2 cells and increased antigen-presenting cell co-stimulation (Figs. 18 and 19).

### 3.4. Biological function disparities between risk groups

**Figure 20.**
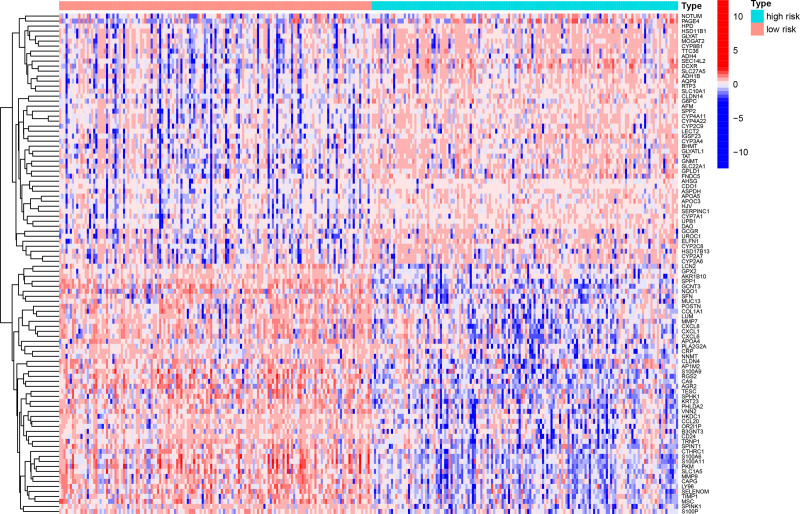
Heatmap of DEGs between high- and low-risk groups. Rows represent genes, columns represent samples grouped by risk, and colors indicate expression *Z*-scores. DEG = differentially expressed gene.

**Figure 21.**
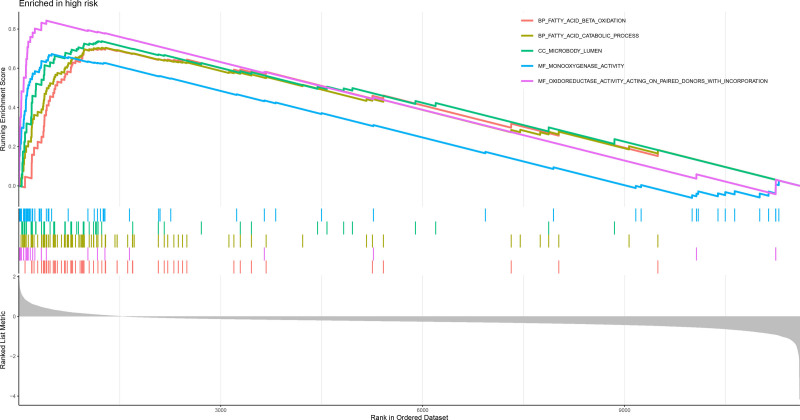
GSEA enrichment plot of GO terms significantly enriched in the high-risk group, including fatty acid oxidation and peroxisome activity. GO = gene ontology, GSEA = gene set enrichment analysis.

**Figure 22.**
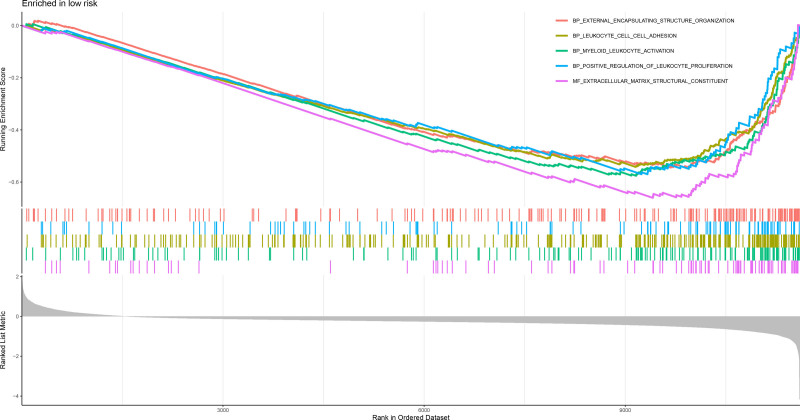
GSEA enrichment plot of GO terms enriched in the low-risk group, highlighting immune cell adhesion and extracellular matrix organization. GO = gene ontology, GSEA = gene set enrichment analysis.

**Figure 23.**
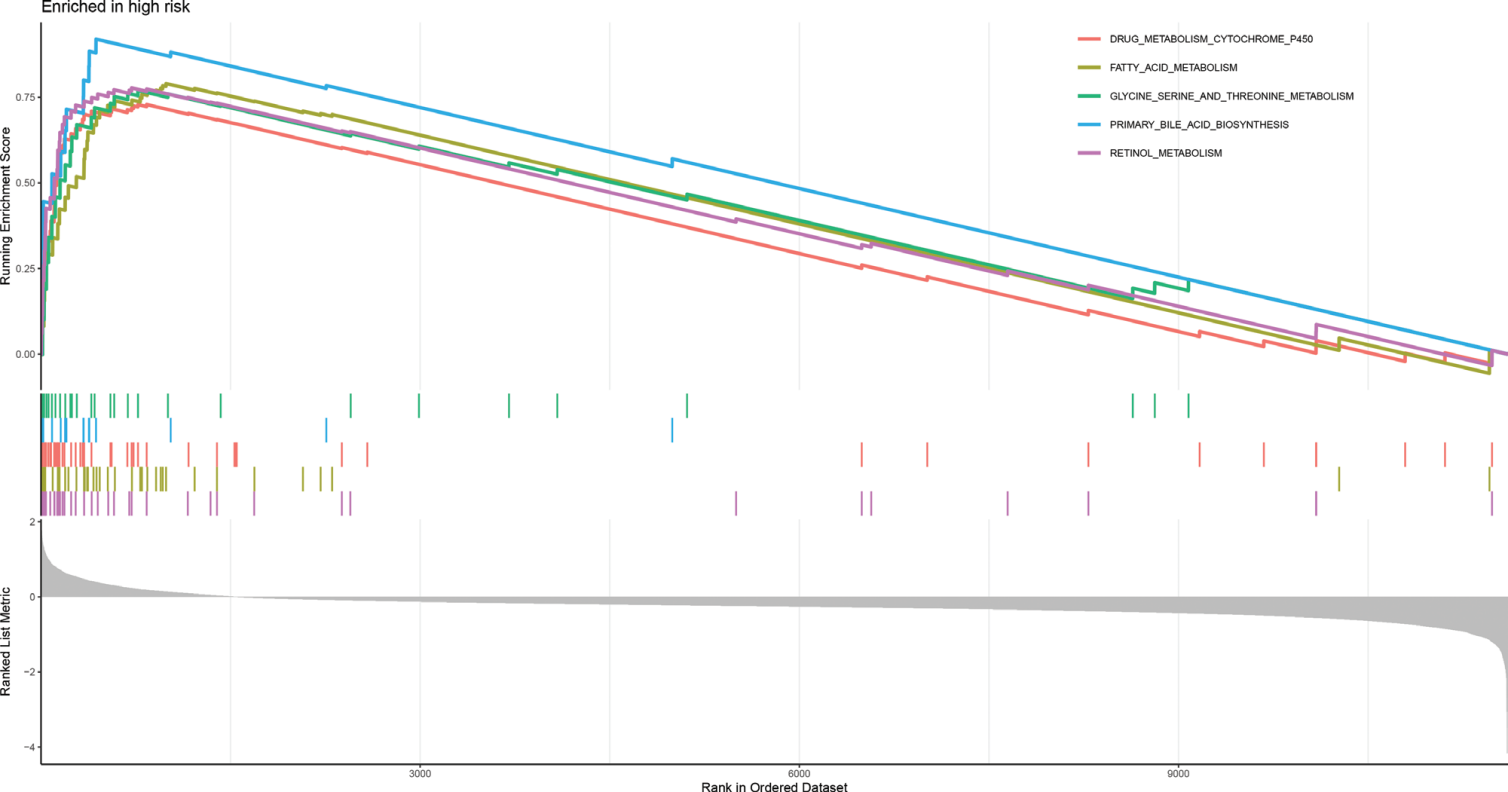
GSEA enrichment plot of KEGG pathways enriched in the high-risk group, such as drug metabolism and fatty acid degradation. GSEA = gene set enrichment analysis, KEGG = kyoto encyclopedia of genes and genomes.

**Figure 24.**
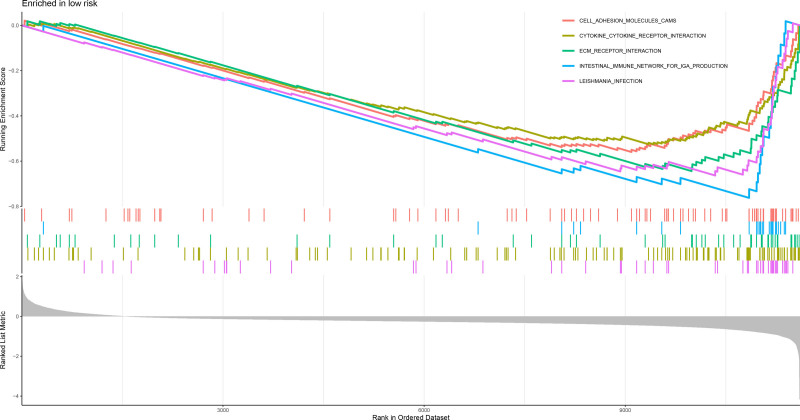
GSEA enrichment plot of KEGG pathways enriched in the low-risk group, including cell adhesion molecules and cytokine–receptor interactions. GSEA = gene set enrichment analysis, KEGG = kyoto encyclopedia of genes and genomes.

Analysis of the 321 DEGs between the risk groups (Fig. 20) revealed distinct functional profiles. GSEA using GO terms revealed low-risk group enrichment in ECM organization, leukocyte adhesion, myeloid cell activation, and leukocyte proliferation, whereas the high-risk group was enriched in fatty acid β-oxidation, peroxisome activity, and oxidoreductase function (Figs. 21 and 22). KEGG-based GSEA revealed low-risk group activity in cell adhesion molecules, cytokine–receptor interactions, and ECM-receptor pathways, whereas the high-risk group exhibited metabolic pathway activation (drug–xenobiotic metabolism, fatty acid–glycine degradation, and bile acid–retinol metabolism; Figs. 23 and 24).

### 3.5. Immunological profiling of core genes

**Figure 25.**
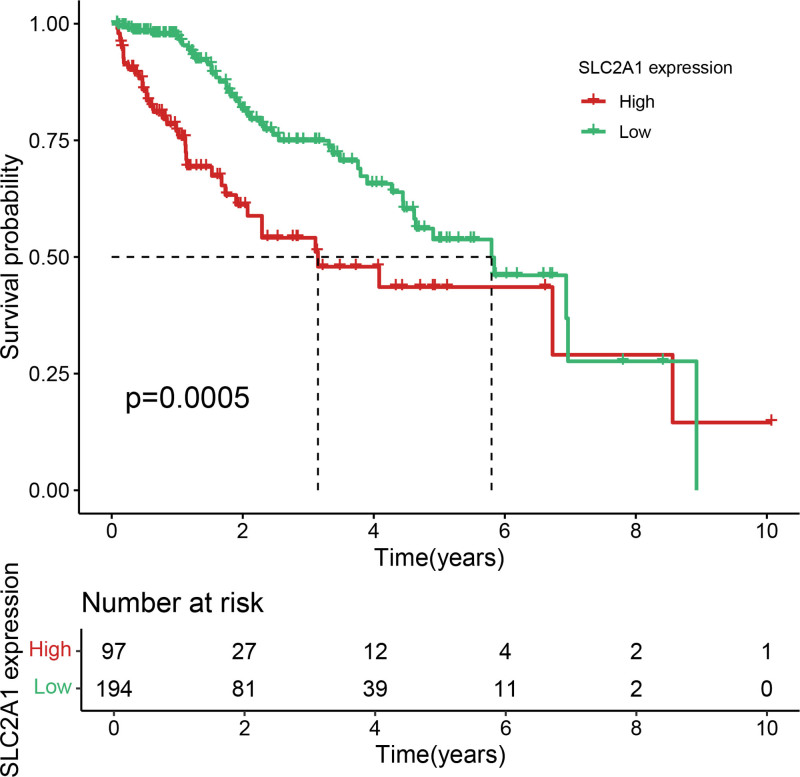
K–M survival curves for OS based on SLC2A1 expression in the TCGA cohort. High expression is associated with poorer survival. K–M = Kaplan–Meier, TCGA = the cancer genome atlas.

**Figure 26.**
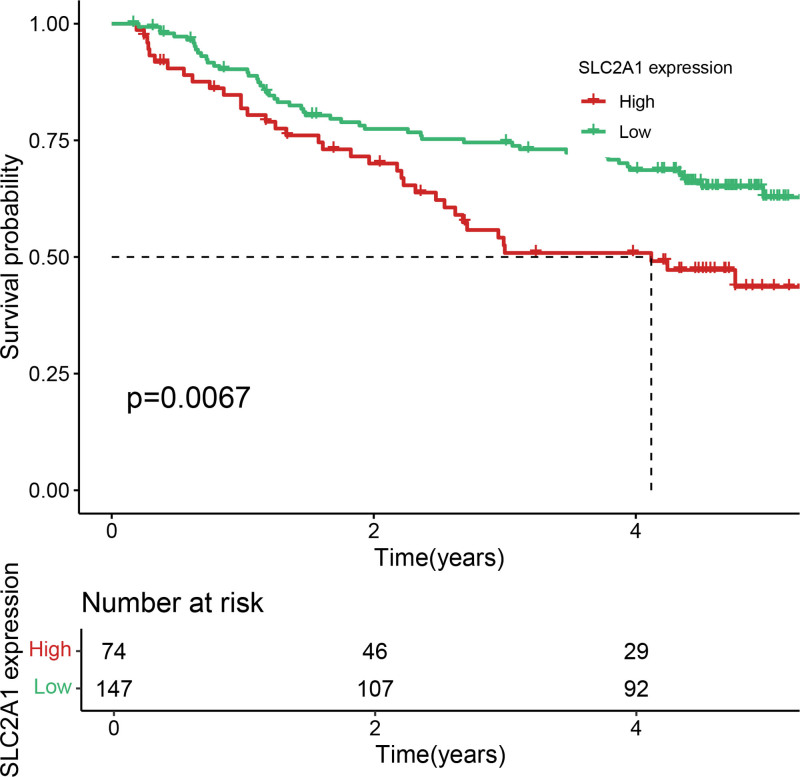
K–M survival curves for OS based on SLC2A1 expression in the GSE14520 cohort, validating its prognostic value. K–M = Kaplan–Meier, OS = overall survival.

**Figure 27.**
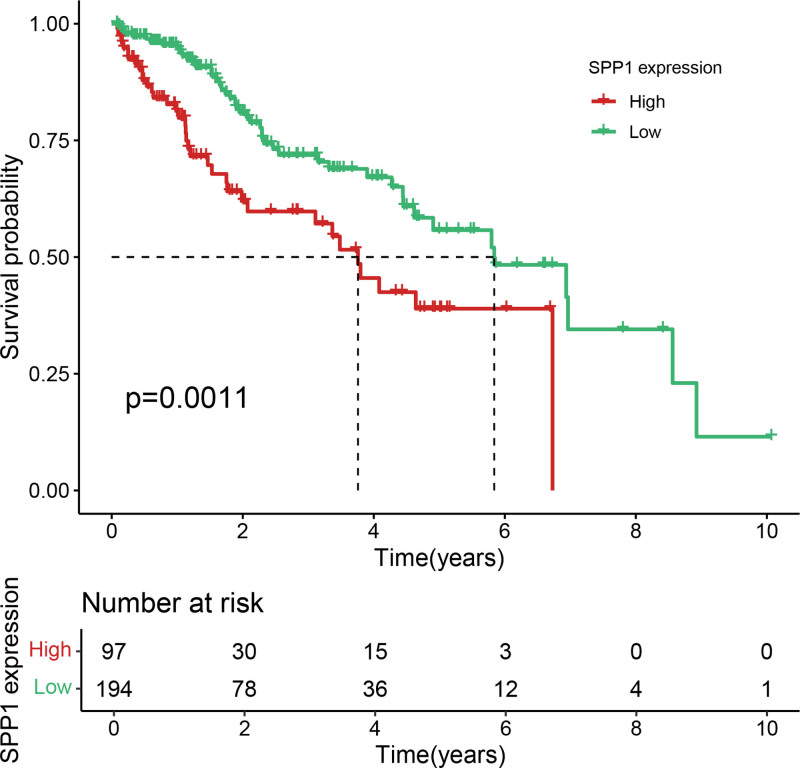
K–M survival curves for OS based on SPP1 expression in TCGA. High SPP1 expression correlates with reduced OS. K–M = Kaplan–Meier, OS = overall survival, TCGA = the cancer genome atlas.

**Figure 28.**
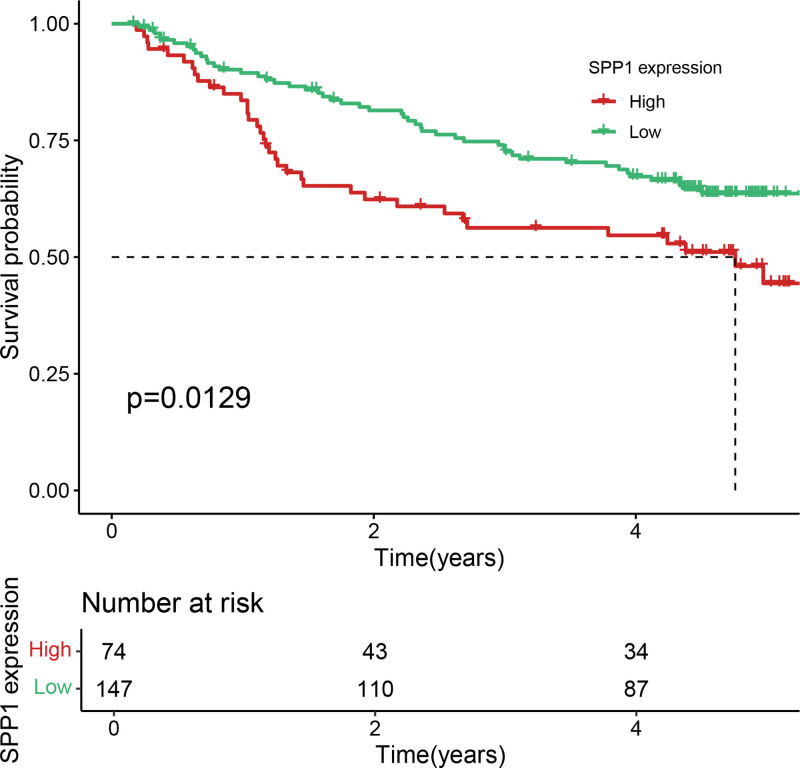
K–M survival curves for OS based on SPP1 expression in GSE14520, confirming its negative prognostic role. K–M = Kaplan–Meier, OS = overall survival.

**Figure 29.**
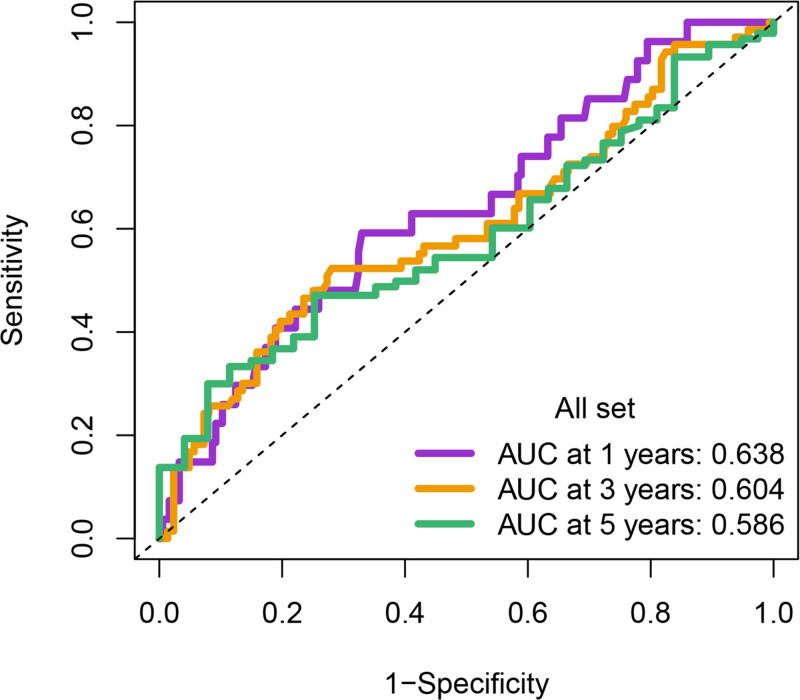
ROC curves for 1-, 3-, and 5-yr OS predictions based on SLC2A1 expression in GSE14520. OS = overall survival, ROC = receiver operating characteristic.

**Figure 30.**
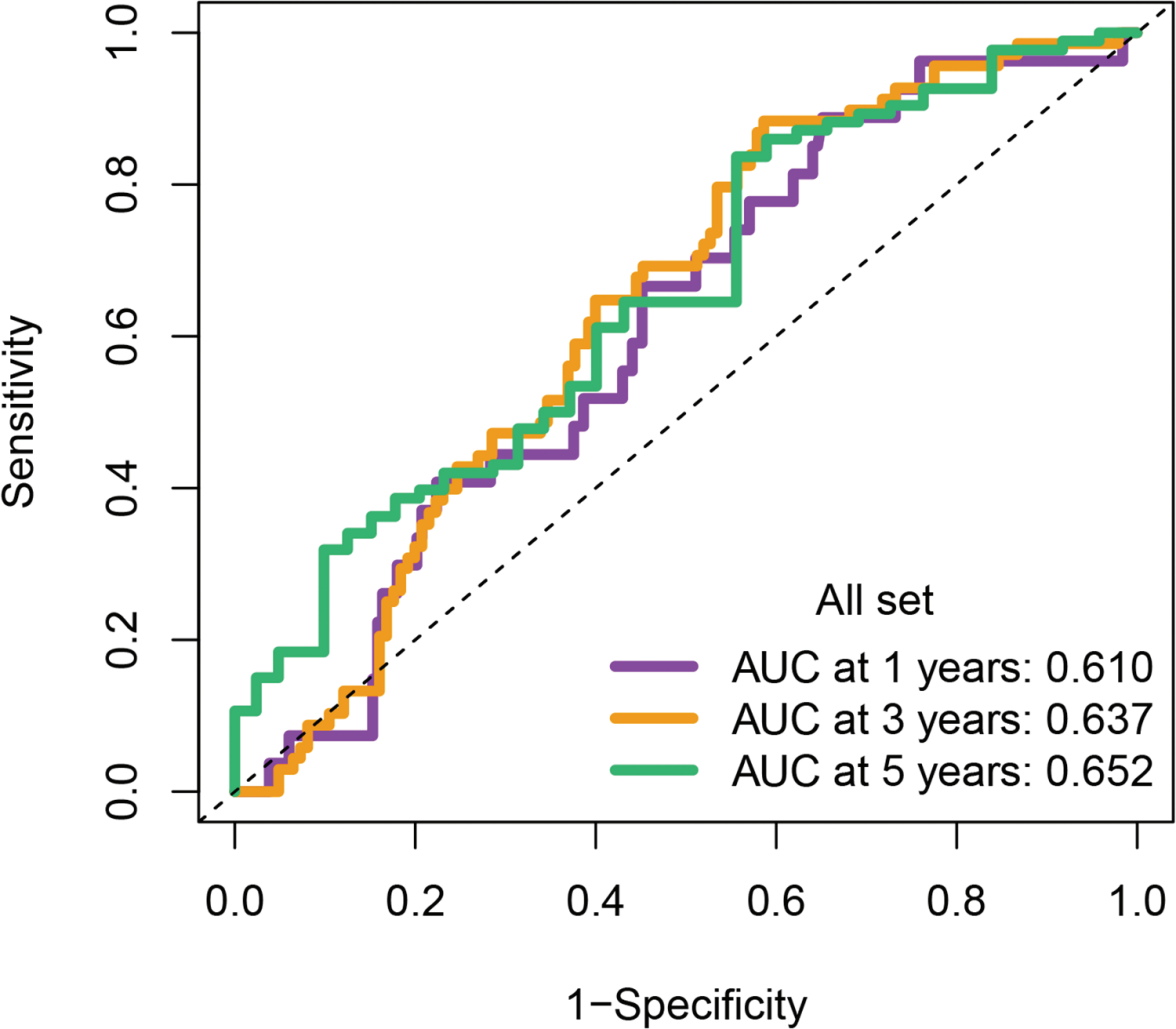
ROC curves for 1-, 3-, and 5-yr OS predictions based on SPP1 expression in GSE14520. OS = overall survival, ROC = receiver operating characteristic.

**Figure 31.**
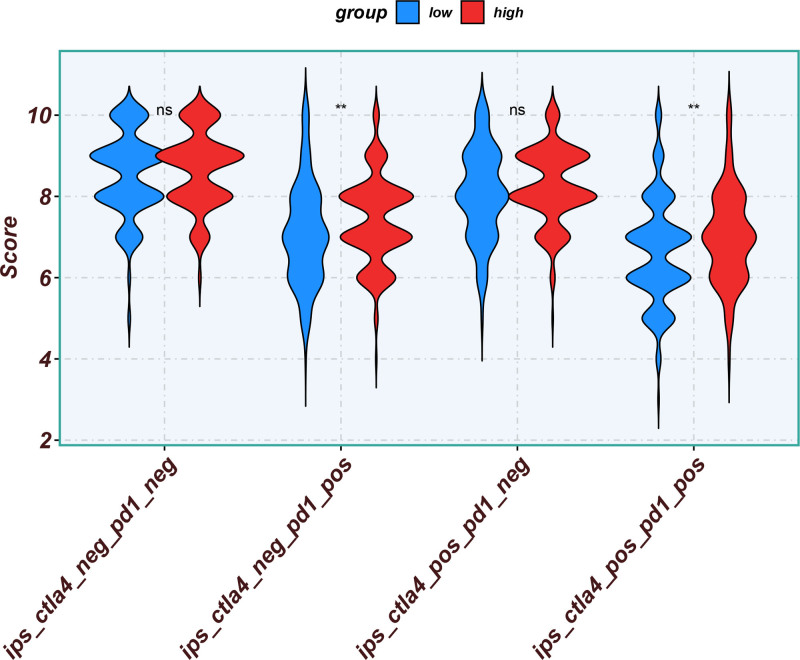
Immunophenoscore (IPS) associated with ITGAM expression. IPS = immunophenoscore.

**Figure 32.**
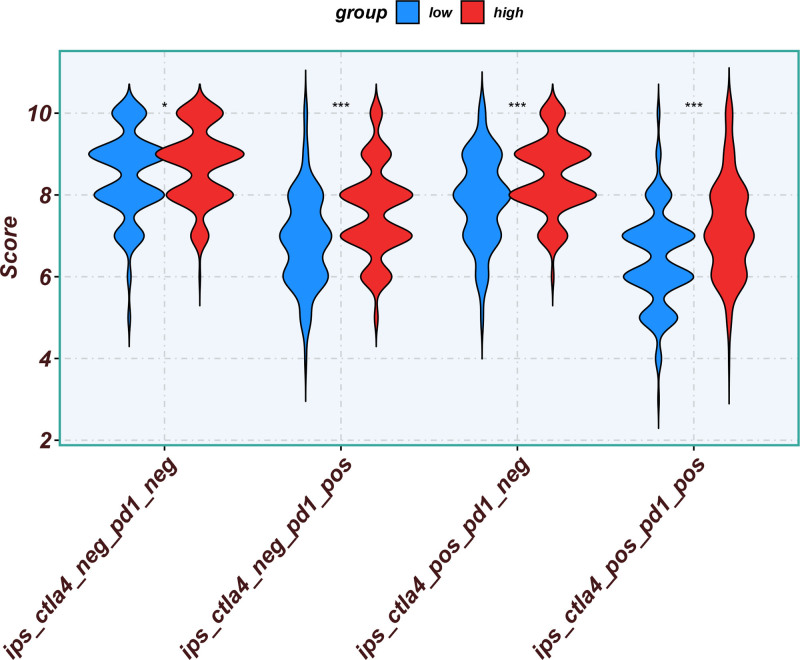
Immunophenoscore (IPS) associated with P2RY6 expression. IPS = immunophenoscore.

**Figure 33.**
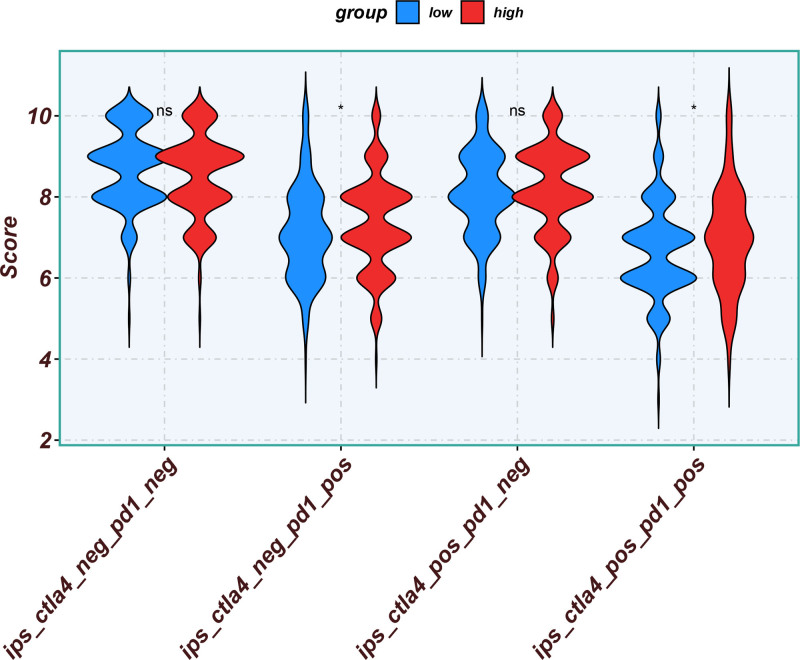
Immunophenoscore (IPS) associated with ST6GALNAC4 expression. IPS = immunophenoscore.

**Figure 34.**
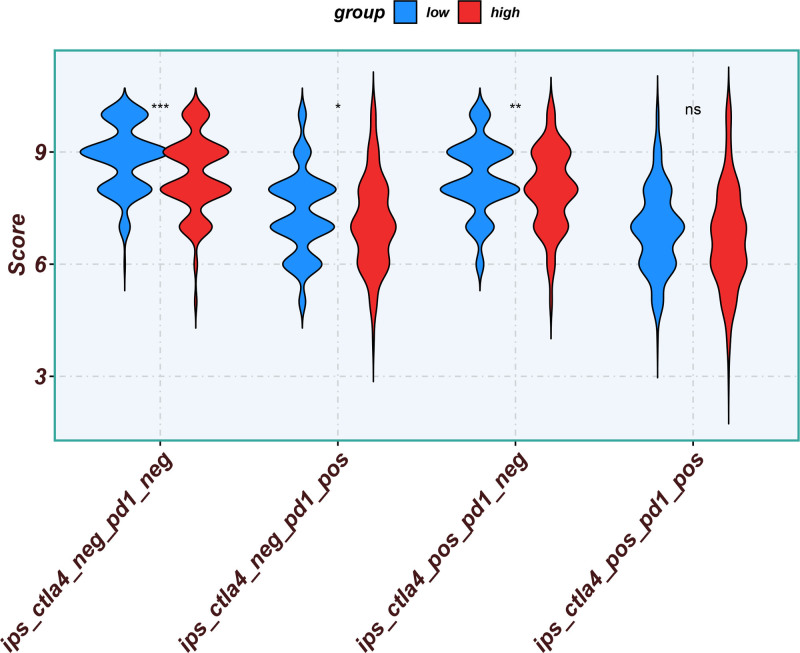
Immunophenoscore (IPS) associated with HILPDA expression. IPS = immunophenoscore.

**Figure 35.**
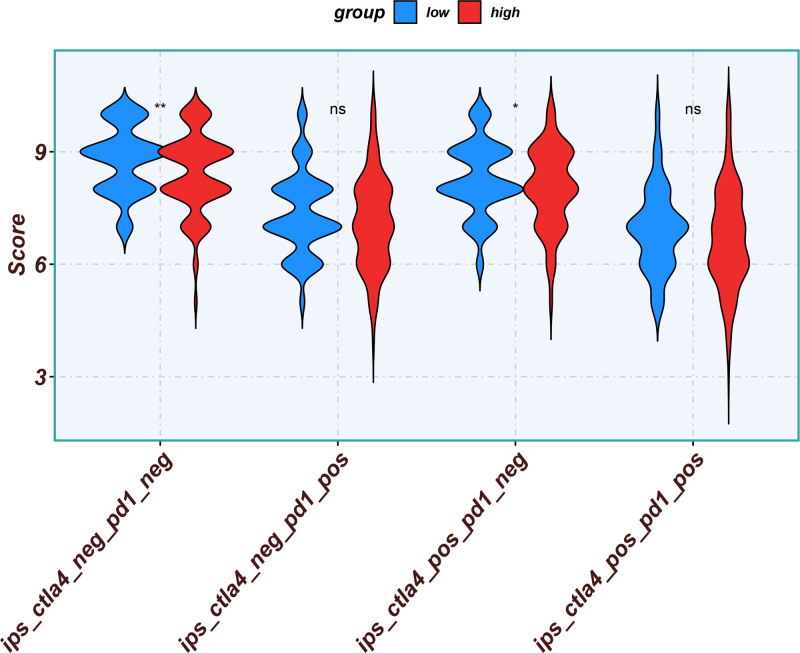
Immunophenoscore (IPS) associated with KIF20A expression. IPS = immunophenoscore.

Eleven core genes (GPC1, HILPDA, ITGAM, KIF20A, P2RY6, PTGS1, SEMA6A, SLC2A1, SPP1, ST6GALNAC4, and UNC5B) were identified through the consensus of the top-performing ML models. Survival analysis confirmed a poor OS in patients with high SLC1A2 (Figs. 25 and 26) and SPP1 expression (Figs. 27 and 28). ROC validation yielded 1-, 3-, and 5-year AUCs of 0.638, 0.604, and 0.586, respectively, for SLC1A2 (Fig. 29), and 0.610, 0.637, and 0.652, respectively, for SPP1 (Fig. 30). IPS analysis revealed increased PD-1/CTLA4 inhibitor sensitivity in patients with high ITGAM (Fig. 31), P2RY6 (Fig. 32), and ST6GALNAC4 (Fig. 33) expression, and low HILPDA (Fig. 34) or KIF20A (Fig. 35) expression.

## 4. Discussion

HCC, which is the fourth leading cause of cancer-related deaths globally and the second leading cause of death among males, remains a critical clinical challenge. According to the surveillance, epidemiology, and end results program,^[22]^ the 5-year survival rate for liver and intrahepatic bile duct cancers is only 20.8%, with localized and distant metastatic cases showing stark disparities (36.1% vs 3.1%). Despite advancements in early detection and therapeutics, outcomes for advanced HCC remain dismal, underscoring the urgent need for novel biomarkers to improve prognosis and reduce the disease burden.^[23]^

In this study, HCC samples were classified into 2 molecular subtypes (A/B) via consensus clustering of chemokine-related genes. Compared to subtype A patients, subtype B patients exhibited significantly superior OS, distinct molecular profiles, pathway activities, and immune infiltration patterns. Functional enrichment analysis revealed differential gene involvement in leukocyte migration, chemotaxis, ECM organization/remodeling, cytochrome P450 metabolism, and Th17/Th1/Th2 cell differentiation. HCC progression arises from chronic inflammation, ECM dysregulation, and metabolic aberrations, and chemokine-driven immune cell infiltration (tumor-associated macrophages and regulatory T cells) exacerbates hepatocyte damage via IL-6/TNF-α secretion and promotes ECM degradation through matrix metalloproteinase (MMP) upregulation,^[24]^ facilitating tumor invasion. Concurrently, excessive ECM deposition activates hepatic stellate cells via integrin-YAP/TAZ signaling, driving EMT and fibrogenic microenvironments.^[25]^ These mechanisms highlight the pivotal regulatory role of chemokine-related genes in HCC.

Recent advances in gene expression-based prognostic models for HCC have focused on neutrophil-derived,^[26]^ glutamine metabolism-related,^[27]^ senescence-associated,^[28]^ and Natural Killer cell heterogeneity-related genes,^[29]^ thereby demonstrating robust predictive utility. This study employed a novel computational framework^[21]^ that integrates 10 machine-learning algorithms and 101 combinatorial models to identify a stable chemokine-related prognostic signature. Validation confirmed its efficacy in risk stratification and independent prognostic value. Although the overall accuracy of the prognostic model is moderate, its high sensitivity – particularly in the validation cohort – suggests clinical utility in reliably identifying patients with poor survival outcomes. This characteristic is valuable for prioritizing high-risk patients for more aggressive surveillance or adjuvant therapy. The relatively lower specificity, especially at longer time points, may reflect the increasing influence of non-tumor factors on survival over time, or inherent heterogeneity within HCC progression. Future models may benefit from incorporating dynamic clinical variables to improve specificity without compromising sensitivity. GSEA revealed biological heterogeneity between risk groups; low-risk patients exhibited enrichment in ECM remodeling, leukocyte regulation, and gut immune networks, suggesting that active immune–stromal crosstalk may suppress metastasis via integrin signaling and modulate the microbiota–liver axis through IgA-mediated immunity. However, excessive myeloid cell activation may paradoxically promote tumorigenesis, necessitating immune subset-specific analysis. In contrast, high-risk patients were enriched in fatty acid β-oxidation, drug metabolism, and oxidoreductase activity, reflecting tumor metabolic reprogramming to sustain proliferation and oxidative stress resistance. Overactivated fatty acid oxidation fuels rapid tumor growth,^[30]^ whereas reactive oxygen specie drive malignant phenotypes via NF-κB activation.^[31]^ These findings suggest that HCC heterogeneity stems from an imbalance between the “immune–stromal homeostasis” and “metabolic-oxidative stress” axes.

Notably, this study revealed a dissociation between the immune scores and functional immune activity. Despite higher immune scores in high-risk patients, their microenvironment is dominated by immunosuppressive cells (regulatory T cells and M2 macrophages) and T cell exhaustion, limiting antitumor immunity. Conversely, low-risk patients demonstrated enhanced immune efficacy through the synergistic effects of activated CD4^+^ T cells (Th1/Tfh subsets) and antigen-presenting cells. These observations emphasize the need to integrate functional biomarkers (costimulatory molecules and T-cell activation status) rather than rely solely on immune scores for immunotherapy stratification.

Cross-model analysis identified 11 core genes (GPC1, HILPDA, ITGAM, KIF20A, P2RY6, PTGS1, SEMA6A, SLC2A1, SPP1, ST6GALNAC4, and UNC5B) with synergistic prognostic values. These genes collectively promote HCC progression and influence the response to immunotherapy by regulating tumor metabolism, the immune microenvironment, and signaling pathways. SPP1 is highly expressed in the HCC microenvironment by tumor-associated macrophages^[32]^ and cancer-associated fibroblasts.^[33]^ Through interactions such as SPP1-CD44, it forms a tumor immune barrier that restricts immune cell infiltration and leads to resistance to immunotherapy.^[34]^ Meanwhile, cancer-associated fibroblasts-derived SPP1 can also activate multiple pro-oncogenic signaling pathways, promoting EMT^[35]^ and hepatic stellate cell activation,^[34]^ thereby driving HCC metastasis and conferring resistance to targeted therapies such as sorafenib and lenvatinib, making it a key factor in poor prognosis and a potential therapeutic target. SLC2A1 (GLUT1) is a key promoter of glycolysis in HCC. Its expression is suppressed by isoginkgetin through CDK6 degradation and inhibition of the AMPK-ULK1 pathway,^[36]^ and is also regulated by lncRNA FTO-IT1^[37]^ and SLC2A1-DT^[38]^ via m6A RNA modification or positive transcriptional regulation by c-Myc, thereby enhancing glucose uptake, glycolysis, and autophagy, ultimately driving HCC progression.

Studies have shown that ST6GALNAC4 promotes tumor proliferation and invasion by inducing abnormal glycosylation of TGFBR2 to activate the TGF-β signaling pathway, while also contributing to the formation of an immunosuppressive microenvironment.^[39]^ HILPDA (HIG2), as a hypoxia-responsive gene, promotes NASH-related HCC development by inhibiting fatty acid metabolism and suppresses NK cell function via the IL-10/STAT3 signaling axis, mediating immune escape.^[40]^ In terms of cell cycle regulation, KIF20A promotes cell proliferation by regulating E2F and G2/M phase signaling, and its overexpression is associated with c-Myc stability and T cell exhaustion.^[41]^ Inhibition of KIF20A significantly enhances the efficacy of anti-PD-1 therapy, suggesting its potential as a target for sensitizing tumors to immunotherapy.^[42]^ GPC1 regulates the proliferation and apoptosis resistance of HCC cells via the Hippo and AKT signaling pathways, and its expression is associated with immune cell infiltration and poor prognosis.^[43,44]^ Furthermore, non-coding RNA-mediated regulatory networks play important roles in HCC. SEMA6A-AS1 affects HCC progression by regulating SEMA6A mRNA stability,^[45]^ while UNC5B-AS1 promotes EMT and tumor metastasis via the miR-4306/KDM2A axis.^[46]^

Our Survival analysis revealed that high SPP1 and SLC1A2 expression levels were associated with reduced OS (*P* < .05), supporting their roles as independent risk factors. While SPP1 exhibited moderate AUC values (0.610–0.652) for 1-, 3-, and 5-year survival predictions, its time-dependent increasing trend highlights its potential utility in long-term prognosis. In contrast, the decrease in the AUC for SLC1A2 (0.638–0.586) suggests that its predictive capacity may be confounded by tumor heterogeneity or therapeutic interventions, necessitating dynamic monitoring. IPS analysis revealed positive correlations between ITGAM, P2RY6, and ST6GALNAC4 expression and PD-1/CTLA-4 inhibitor sensitivity, whereas low HILPDA and KIF20A expression predicted favorable responses. Mechanistically, KIF20A overexpression competitively inhibited FBXW7-mediated c-Myc degradation, promoting glycolysis and proliferation.^[42]^ Its suppression reduces c-Myc-driven DNA mismatch repair (MMR) protein expression, potentially enhancing PD-1 inhibitor efficacy by increasing tumor antigen diversity. ST6GALNAC4 promotes immunosuppression through the T antigen-galectin 3+ TAM axis,^[39]^ whereas HILPDA promotes HCC survival by inhibiting hypoxia-induced apoptosis.^[40]^ ITGAM (an integrin family member) recruits immunosuppressive macrophages,^[47]^ and P2RY6-expressing TAMs drive immune exclusion and therapy resistance.^[48]^

Although our IPS analysis indicated that elevated expression of ITGAM, P2RY6, and ST6GALNAC4, as well as reduced expression of HILPDA and KIF20A, might predict improved responsiveness to PD-1/CTLA-4 inhibitors, the corresponding survival curves derived from the TCGA and gene expression omnibus cohorts did not uniformly demonstrate poorer outcomes in high-expression groups. This apparent inconsistency may be attributed to the absence of detailed immunotherapy treatment records in these public datasets. Thus, the IPS-derived predictions reflect a theoretical estimation of immunotherapy sensitivity based on transcriptomic patterns rather than actual clinical response outcomes. Further validation in prospective, immunotherapy-treated HCC cohorts is essential to confirm the practical utility of these chemokine-related genes in predicting immunotherapeutic benefits.

Despite outperforming several existing prognostic models for HCC, our approach still has limitations when compared to certain ML-based frameworks. The mechanisms underlying chemokine-related genes and their clinical applicability require further verification through multicenter prospective studies. Future research should focus on: spatial transcriptomic characterization of immune–stromal crosstalk, functional validation of core genes such as SPP1 and ITGAM using organoid or murine models, and the development of metabolism-targeted therapies for high-risk patient subgroups. In summary, this study proposes a novel biomarker framework for prognostic stratification and immunotherapy optimization in HCC, though its clinical translation will necessitate multi-omics integration and functional assays.

## 5. Conclusions

In this study, we established a robust chemokine-centric ML framework that effectively stratifies HCC patients into 2 distinct molecular subtypes with significant differences in OS, immune landscape, and metabolic activity. Through integrative analysis of multi-omics data, we identified a prognostic signature comprising 11 core genes, among which SPP1 and SLC2A1 were strongly associated with poor prognosis, while ITGAM, P2RY6, ST6GALNAC4, HILPDA, and KIF20A showed promise as predictors of response to ICIs. Future efforts should focus on validating these targets in experimental models and prospective clinical cohorts, particularly in the context of immunotherapy-treated HCC patients. Additionally, integrating spatial transcriptomics and single-cell analyses could further elucidate the cellular interactions underpinning the chemokine-immune-metabolic axis, paving the way for personalized therapeutic interventions.

In summary, this study provides a computational and biological foundation for leveraging chemokine signaling networks to improve prognostic accuracy and immunotherapy responsiveness in HCC, with meaningful translational potential.

## Acknowledgments

We acknowledge the contributions of TCGA, GEO, and MSigDB in providing open-access datasets and resources.

## Author contributions

**Conceptualization:** Hao Gu.

**Data curation:** Jinlong Zhou, Xinrong Wei, Cheng Liu.

**Investigation:** Junjie Liu.

**Methodology:** Hao Gu.

**Resources:** Junjie Liu, Cheng Liu.

**Software:** Junjie Liu, Xinrong Wei.

**Supervision:** Hao Gu.

**Validation:** Jinlong Zhou.

**Visualization:** Jinlong Zhou.

**Writing – original draft:** Jinlong Zhou.

**Writing – review & editing:** Hao Gu.
